# Identification of the global diurnal rhythmic transcripts, transcription factors and time-of-day specific *cis* elements in *Chenopodium quinoa*

**DOI:** 10.1186/s12870-023-04107-z

**Published:** 2023-02-16

**Authors:** Qi Wu, Xue Bai, Yiming Luo, Li Li, Mengping Nie, Changying Liu, Xueling Ye, Liang Zou, Dabing Xiang

**Affiliations:** Key Laboratory of Coarse Cereal Processing, Ministry of Agriculture and Rural Affairs, Sichuan Engineering and Technology Research Center of Coarse Cereal Industralization, College of Food and Biological Engineering, Chengdu University, Chengluo Road 2025, Longquanyi District, Chengdu, 610106 Sichuan China

**Keywords:** Diurnal rhythmic genes, Photoperiod, Phase shift, Transcription factors, *Cis* elements, *Chenopodium quinoa*

## Abstract

**Background:**

Photoperiod is an important environmental cue interacting with circadian clock pathway to optimize the local adaption and yield of crops. Quinoa (*Chenopodium quinoa*) in family Amaranthaceae has been known as superfood due to the nutritious elements. As quinoa was originated from the low-latitude Andes, most of the quinoa accessions are short-day type. Short-day type quinoa usually displays altered growth and yield status when introduced into higher latitude regions. Thus, deciphering the photoperiodic regulation on circadian clock pathway will help breed adaptable and high yielding quinoa cultivars.

**Results:**

In this study, we conducted RNA-seq analysis of the diurnally collected leaves of quinoa plants treated by short-day (SD) and long-day conditions (LD), respectively. We identified 19,818 (44% of global genes) rhythmic genes in quinoa using HAYSTACK analysis. We identified the putative circadian clock architecture and investigated the photoperiodic regulatory effects on the expression phase and amplitude of global rhythmic genes, core clock components and transcription factors. The global rhythmic transcripts were involved in time-of-day specific biological processes. A higher percentage of rhythmic genes had advanced phases and strengthened amplitudes when switched from LD to SD. The transcription factors of CO-like, DBB, EIL, ERF, NAC, TALE and WRKY families were sensitive to the day length changes. We speculated that those transcription factors may function as key mediators for the circadian clock output in quinoa. Besides, we identified 15 novel time-of-day specific motifs that may be key *cis* elements for rhythm-keeping in quinoa.

**Conclusions:**

Collectively, this study lays a foundation for understanding the circadian clock pathway and provides useful molecular resources for adaptable elites breeding in quinoa.

**Supplementary Information:**

The online version contains supplementary material available at 10.1186/s12870-023-04107-z.

## Background

Plants sense the fluctuation of environmental cues and utilize circadian clock-based molecular network to fine-tune internal physiological activities [1]. Circadian clock is involved in various biological activities, such as responses to abiotic stresses, leaf movement, stomatal opening and floral transition [2]. The molecular architecture and regulatory network of circadian clock have been intensively investigated in the model plant *Arabidopsis thaliana*. The core circadian clock is composed of several key components-the morning-phased *CIRCADIAN CLOCK ASSOCIATED 1* (*CCA1*) and *LATE ELONGATED HYPOCOTYL* (*LHY*) and the evening-phased *PSEUDO RESPONSE REGULATOR 1* (*PRR1*/*TOC1*), forming inter-repressed feedback regulatory loop [3]. Besides, *PRR3*/*5*/*7*/*9*, the evening complex (EC, EARLY FLOWERING3-ELF4-LUX ARRHYTHMO (ELF3-ELF4-LUX), and *GIGANTEA* (*GI*), respectively, form additional interlocked loops with *CCA1*, *LHY* and *TOC1* [4–7]. Those clock components further regulate thousands of downstream rhythmic transcripts in time-of-day specific manner.

Circadian clock is entrained by daily and seasonal light signal changes [8]. The light signals, including light intensity and length (photoperiod), input to the clock is dependent on various wavelength photoreceptors, such as red/far-red light sensors *PHOTOCHROME A* (*PHYA*) to *PHYE*, blue light receptors *CRYPTOCHROME 1/2* (*CRY1/2*) and *ZEITLUPE* (*ZTL*)/*FKF1*/*LKP2* family [9]. Then the modulation on circadian clock by light signals is transmitted to output cascades via several key mediators such as *CONSTANS* (*CO*), *CYCLING DOF FACTOR* (*CDF*) and *FLOWERING LOCUS T* (*FT*) in photoperiodic flowering, *PHYTOCHROME INTERACTING FACTOR4*/*5* (*PIF4*/*5*) in hypocotyl growth, and *C-REPEAT BINDING FACTOR 1*/*2*/*3* (*CBF1*/*2*/*3*) in cold acclimation [1]. Circadian clock together with light signals have gating effects on the expression of downstream genes at transcriptional and post-transcriptional levels, resulting in altered growth status under different photoperiods [9]. Many evidences in crop plants suggested that modulating the components of circadian clock pathway is an effective way to enhance the local adaption and abiotic resistance, thus enhancing agricultural productivity [10–14].

Quinoa (*Chenopodium quinoa*) has been known as superfood crop originated from the low-latitude Andes. Since the declaration of International Year of Quinoa in 2013, it has been cultivated world widely. Yet, it is still an orphan crop owing to less domestication, and generating adaptable and high yielding quinoa cultivars has become the major breeding target [15]. Short-day quinoa always displays prolonged growth and yield alteration when introduced into higher latitude regions [16–18]. Thus, understanding the circadian clock architecture and regulatory network of quinoa will potentially help breed elite quinoa cultivars.

To date, while plenty of knowledge is unraveled about the photoperiodic regulation on circadian clock in model plants such as Arabidopsis and rice (*Oryza saltiva*), little is known about how quinoa adjusts the internal clock and the global transcription levels in response to day length changes. In this study, we conducted RNA-seq analysis of the diurnally collected leaves of quinoa plants under short-day (SD) and long-day conditions (LD). We identified the putative circadian clock components and investigated the effects of photoperiod on global rhythmic gene expression. SD induced more rhythmic transcripts than LD did. The global rhythmic transcripts were involved in time-of-day specific biological processes. We found a higher percentage of rhythmic genes had advanced phases and strengthened amplitudes when switched from LD to SD. Meanwhile, we found that transcription factors CO-like, DBB, EIL, ERF, NAC, TALE and WRKY were sensitive to the day length changes, and may function as key mediators for the circadian clock output in quinoa. Further, we identified several conserved and 15 novel time-of-day specific motifs that may be key *cis* elements for rhythm-keeping in quinoa. Collectively, this study lays a foundation for understanding the circadian clock pathway and provides useful molecular information for adaptable and high yielding cultivars breeding in future.

## Results

### RNA-seq analysis of the quinoa leaves under long-day and short-day conditions

We compared the phenotypes of quinoa plants grown under short-day (SD) and long-day (LD), and found that different photoperiods have obviously distinct effects on quinoa development, such as bolting date, branch number and plant height. SD obviously accelerated quinoa flowering than LD (Figure S1). To investigate how photoperiod influences time-of-day specific transcripts abundance, quinoa seedlings were treated by LD and SD conditions, respectively, in controlled growth chamber. The top-fully-expanded leaves of 3 ~ 5 plants were collected at Zeitgeber Time 02 (ZT02), ZT05, ZT08, ZT11, ZT14, ZT17, ZT20 and ZT23, over 24 h under LD and SD, respectively. By transcriptome sequencing, an average of 25.08 million PE150 clean reads were generated for each sample. In total, 240 Gb clean data for 32 samples was yielded (Table 1). The average Q30 value reached 93% (Table 1). Subsequently, the clean reads were mapped to quinoa reference genome [19] by using HISAT2 [20], and all the mapping rates were above 96% (Table 1). Then the gene expression levels were normalized to fragments per kilo base of transcript per million fragments mapped (FPKM) value by applying StringTie [21]. Thus, the time-course gene expression data over 24 h for LD and SD was obtained, respectively.

**Table 1:** Summary of RNA-seq data of samples collected under SD and LD

Time point		Clean reads	Clean bases	Q30 percentage	Total Reads	Mapped Reads
**LD**	ZT02-1	26,321,633	7,871,952,920	93.19%	52,643,266	51,053,313 (96.98%)
	ZT02-2	21,882,598	6,549,001,050	94.15%	43,765,196	42,580,250 (97.29%)
	ZT05-1	27,084,674	8,104,361,492	93.51%	54,169,348	52,580,003 (97.07%)
	ZT05-2	28,649,148	8,568,024,658	93.56%	57,298,296	55,575,024 (96.99%)
	ZT08-1	32,066,048	9,589,583,626	93.74%	64,132,096	62,244,194 (97.06%)
	ZT08-2	28,663,037	8,574,125,150	93.74%	57,326,074	55,743,730 (97.24%)
	ZT11-1	30,677,275	9,177,060,916	93.65%	61,354,550	59,577,873 (97.10%)
	ZT11-2	23,983,084	7,175,595,724	93.29%	47,966,168	46,581,179 (97.11%)
	ZT14-1	26,925,971	8,052,505,520	93.92%	53,851,942	52,318,593 (97.15%)
	ZT14-2	25,417,823	7,599,257,510	93.70%	50,835,646	49,323,300 (97.03%)
	ZT17-1	33,457,625	10,010,422,544	93.55%	66,915,250	64,968,515 (97.09%)
	ZT17-2	20,766,746	6,212,860,922	93.75%	41,533,492	40,333,702 (97.11%)
	ZT20-1	23,430,876	7,011,487,036	93.87%	46,861,752	45,460,250 (97.01%)
	ZT20-2	29,659,011	8,873,295,840	93.55%	59,318,022	57,527,939 (96.98%)
	ZT23-1	31,512,284	9,424,322,988	93.37%	63,024,568	61,050,636 (96.87%)
	ZT23-2	22,580,283	6,756,594,052	93.77%	45,160,566	43,796,867 (96.98%)
**SD**	ZT02-1	23,199,936	6,941,939,276	92.91%	46,399,872	44,736,757 (96.42%)
	ZT02-2	21,872,672	6,544,191,570	92.59%	43,745,344	42,160,350 (96.38%)
	ZT05-1	20,817,060	6,226,933,522	93.15%	41,634,120	40,250,667 (96.68%)
	ZT05-2	21,504,896	6,434,810,038	92.91%	43,009,792	41,594,910 (96.71%)
	ZT08-1	26,923,663	8,051,025,032	93.63%	53,847,326	52,259,506 (97.05%)
	ZT08-2	24,018,373	7,180,476,390	93.97%	48,036,746	46,662,046 (97.14%)
	ZT11-1	23,258,589	6,962,452,472	92.94%	46,517,178	44,928,284 (96.58%)
	ZT11-2	22,872,893	6,848,013,940	93.48%	45,745,786	44,304,932 (96.85%)
	ZT14-1	21,886,436	6,552,647,046	93.68%	43,772,872	42,433,602 (96.94%)
	ZT14-2	27,629,362	8,263,889,294	93.40%	55,258,724	53,441,744 (96.71%)
	ZT17-1	22,250,747	6,660,876,010	93.63%	44,501,494	43,037,893 (96.71%)
	ZT17-2	23,954,561	7,171,762,348	93.36%	47,909,122	46,271,953 (96.58%)
	ZT20-1	19,989,579	5,981,657,792	92.18%	39,979,158	38,445,139 (96.16%)
	ZT20-2	24,799,448	7,410,007,430	92.77%	49,598,896	47,778,300 (96.33%)
	ZT23-1	22,245,857	6,644,693,296	92.35%	44,491,714	42,881,649 (96.38%)
	ZT23-2	22,388,748	6,701,270,476	92.34%	44,777,496	43,184,110 (96.44%)

### HAYSTACK analysis indicates 44% of the global transcripts are diurnally rhythmic

**Fig. 1:**
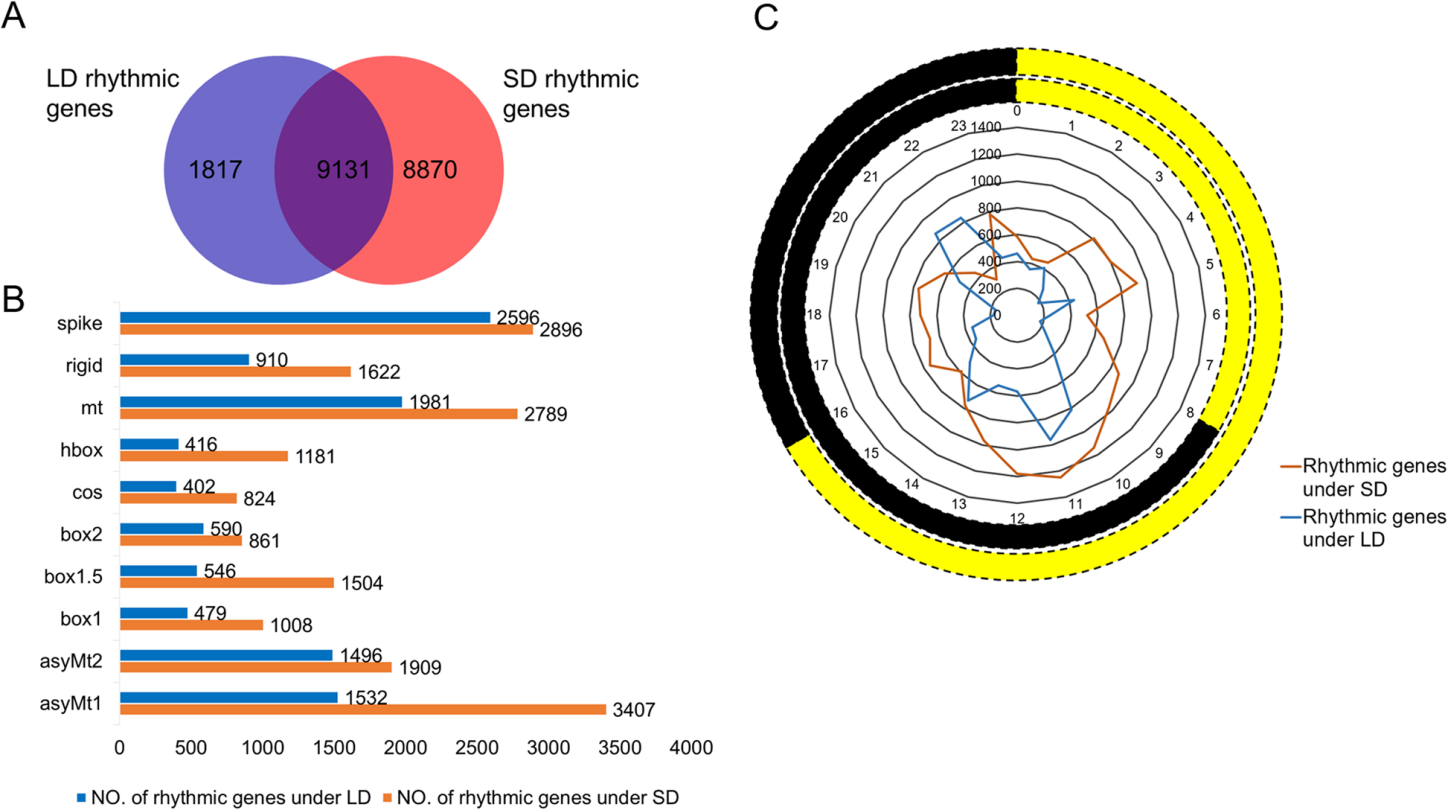
44.26% of the global transcripts were identified as diurnal cycling genes in quinoa by using HAYSTACK algorithm. **A** Venn diagram of the rhythmic genes under SD and LD. 20.39% of the global transcripts displayed rhythmicity under both photoperiods. 19.81% and 4.05% were SD-specific and LD-specific oscillating, respectively. **B** Statistics of the cycling transcripts classified into different models. Spike model and Asymt1 model harbored the most genes under LD and SD, respectively, and Cos model contained the least cycling genes regardless of light length. **C** Numbers of cycling transcripts phased to different time points (ZT00 to ZT23). The outer two cycles represent light conditions under SD and LD. Yellow and black stand for day and night time, respectively

To identify the rhythmic genes under different photoperiods in quinoa, we submitted the transcription profiles to HAYSTACK website. HAYSTACK algorithm is a powerful method for identifying cycling genes [22], which has been successfully applied to detect rhythmic oscillating transcripts in many plants, such as *Arabidopsis thaliana*, pineapple (*Ananas comosus*), rice (*Oryza saltiva*), and poplar (*Populus trichocarpa*) [22–24]. To conduct HAYSTACK analysis, we developed a set of models covering ZT02, ZT05, ZT08, ZT11, ZT14, ZT17, ZT20 and ZT23 time points. Totally, 636 non-redundant models containing Asymt1, Asymt2, Box1, Box1.5, Box2, Cos, hBox, Mt, Rigid and Spike-like patterns, derived from the predefined 1 h-resolution models [23, 25], were adopted for rhythmicity determination in this study (Figure S2, Table S1). Based on the criteria that Pearson correlation cutoff ≥ 0.8, fold change of largest value/smallest value ≥ 2, background FPKM cutoff ≥ 1.0 and *p*-value ≤ 0.01, 10,948 and 18,001 diurnal cycling genes were identified from LD and SD time-serial data (Fig. 1, Tables S2 and S3), respectively. About 44.26% (19,818 out of 44,776) of the global transcripts have rhythmic expression patterns under at least one photoperiod, and 20.39% (9131) of the total genes displayed rhythmicity under both LD and SD (Fig. 1A). We found 19.81% (8870) of the total genes were SD-specific oscillating, whereas the LD-specific oscillating genes percentage decreased to 4.05% (Fig. 1A), indicating day-length-specific diurnal regulation.

According to the HAYSTACK analysis results, cycling genes were more abundant in Spike model (2596) under LD, whereas Asymt1 model (3407) harbored more genes when switched to SD (Fig. 1B, Tables S2 and S3). Cos-type transcripts were the least under both LD and SD (Fig. 1B, Tables S2 and S3). Meanwhile, HAYSTACK analysis classified the rhythmic transcripts into 24 phase bins according to the peak expression time (Fig. 1C, Tables S2 and S3). As to LD-treated plants, cycling genes were more abundant at the late of day (ZT11) and night (ZT21, ZT22) periods, respectively (Fig. 1C). When transferred to SD, a larger proportion of cycling genes had peak expression at early night time (ZT10 to ZT12) (Fig. 1C). Notably, more diurnal cycling genes phased to the middle time over entire day regardless of the day length (Fig. 1C).

### Rhythmic genes are involved in time-of-day specific biological processes

**Fig. 2:**
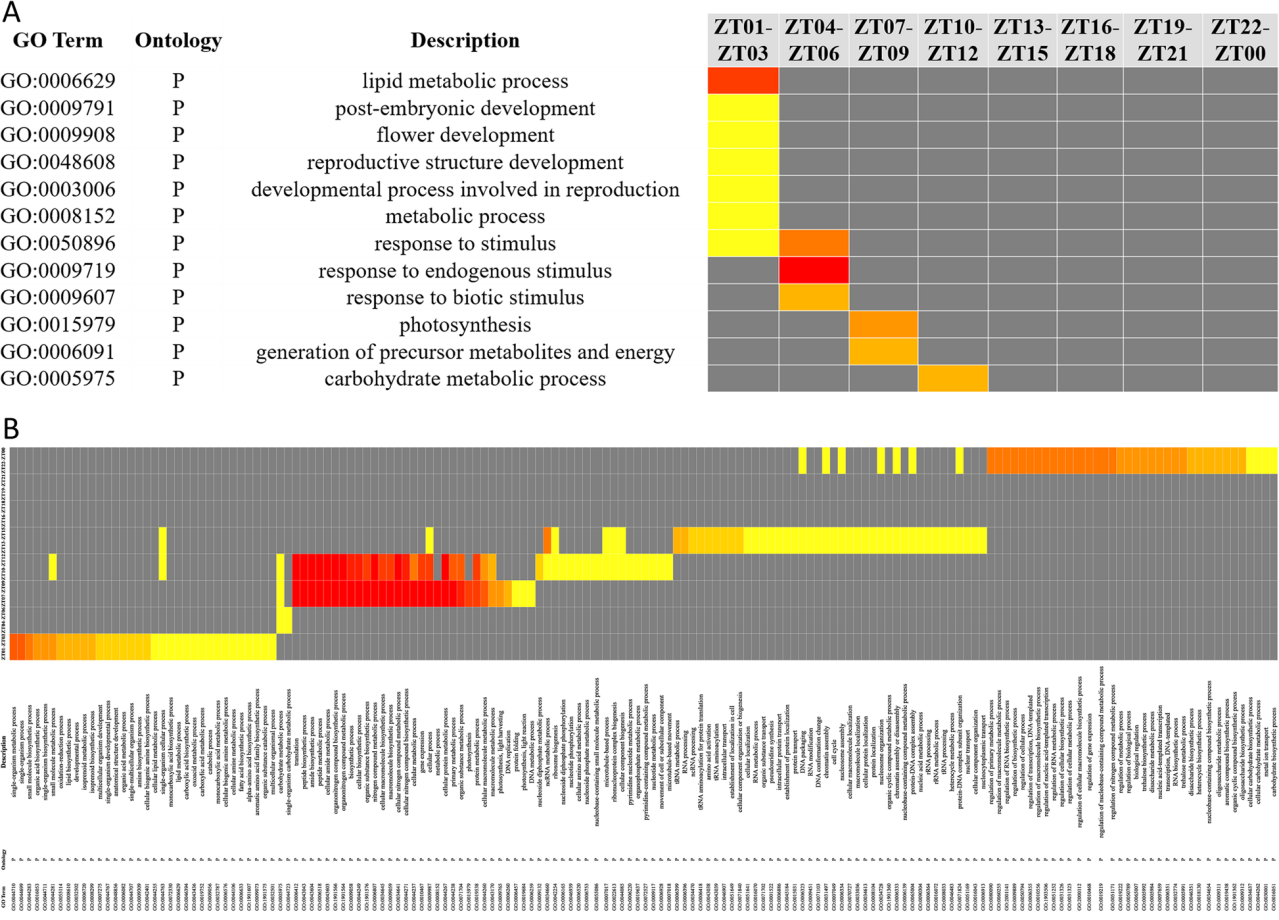
The diurnal rhythmic genes were enriched in time-of-day specific biological processes. Gene ontology analysis of rhythmic genes under LD (**A**) and SD (**B**) was performed using AgriGO v2.0 tool with False Discovery Rate (FDR) ≤ 0.05. Red indicates more significant enriched items

To understand what internal biological activities that quinoa adjusts in response to photoperiod changes, gene ontology (GO) analysis was performed to investigate the biological processes of different phase bin genes under LD and SD, respectively. We used AgriGO tool [26] to interpret the functions of rhythmic genes in three consecutive phase bins. Most rhythmic genes displayed time-of-day specific enrichment patterns. Under LD, several metabolism and development relevant terms, such as “lipid metabolic process”, “post-embryonic development”, “flower development”, “reproductive structure development”, “developmental process involved in reproduction” and “metabolic process”, were specifically overrepresented at the beginning of light period (Fig. 2A). GO terms like “response to stimulus”, “response to endogenous stimulus” and “response to biotic stimulus” were specifically phased to ZT04 to ZT06, while “photosynthesis” and “carbohydrate metabolic process” were significantly enriched in ZT07 to ZT09 and ZT10 to ZT12, respectively (Fig. 2A). There were no terms enriched for cycling genes phased to the late time of light period and entire dusk period (Fig. 2A).

Compared with the number of enriched terms under LD, more terms were overrepresented for SD cycling transcripts. “Lipid metabolic process”, and many small molecule metabolic processes, such as “organic acid biosynthetic process”, “isoprenoid metabolic process”, “amine biosynthetic process”, “carboxylic acid metabolic process” and “fatty acid biosynthetic process”, were phased to the beginning of light period (ZT01 to ZT03) (Fig. 2B). The genes phased to ZT04 to ZT12 were all involved in “carbohydrate metabolic process” (Fig. 2B). At the end of light period (ZT07 to ZT09), “photosynthesis, light harvesting, and light reaction”, “DNA metabolic process” and “protein folding” associated terms became specifically significant. Transcripts phased to the end of light period (ZT07 to ZT09) and the beginning of dusk period (ZT10 to ZT12) were highly represented in “nitrogen compound metabolic process” and “protein metabolic process” related terms (Fig. 2B). In the middle of dusk period, more cycling genes were highly associated with “RNA, tRNA, rRNA processing, modification” and “macromolecule localization” related terms (Fig. 2B). At dawn time, many transcriptional regulatory terms, including “regulation of gene expression”, “regulation of RNA biosynthetic process” and “RNA biosynthetic process”, were overrepresented (Fig. 2B).

### Photoperiodic effects on the phase and amplitude of diurnal rhythmic transcripts

**Fig. 3:**
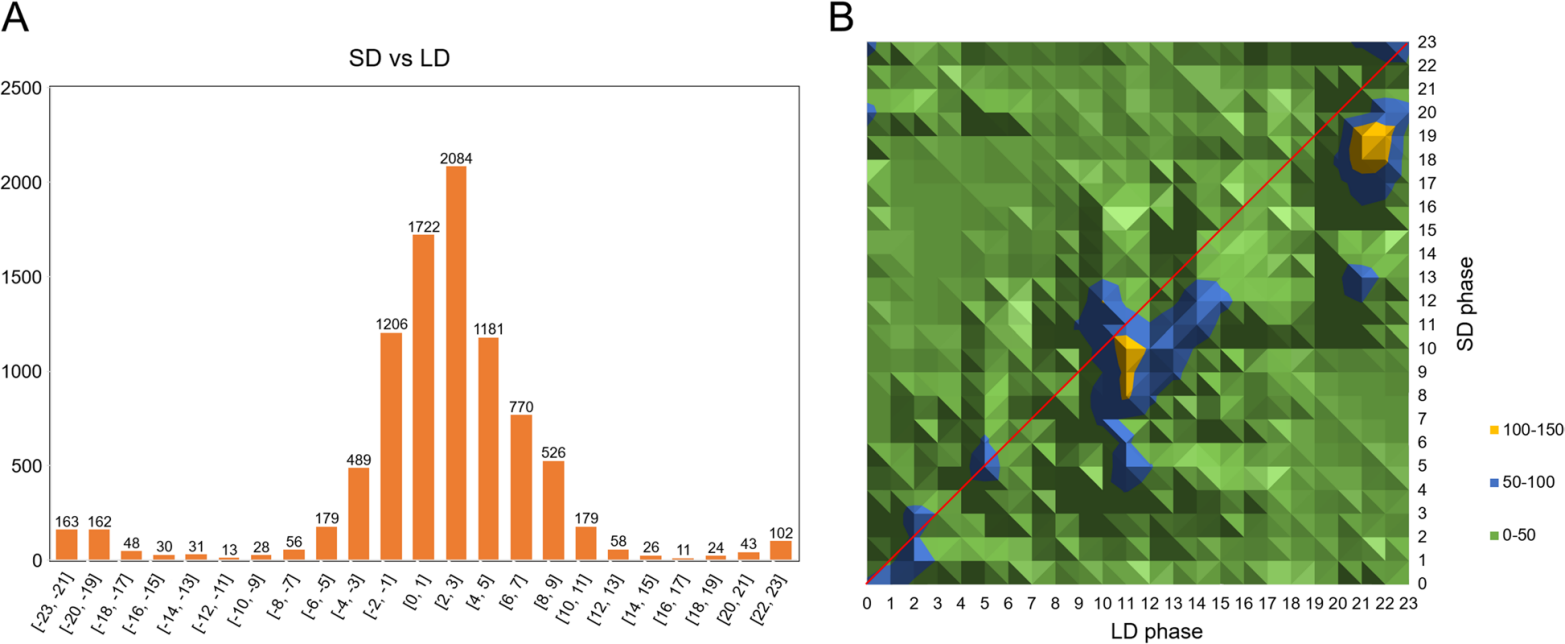
Comparison of the phases of oscillating transcripts between SD and LD. **A** Numbers of transcripts that phase-shifted to different hours between SD and LD. SD is the reference. **B** Phase shift topology map of the oscillating transcripts between SD and LD. Only the cycling genes displayed rhythm both under SD and LD were used for phase shift analysis

To optimize the growth and development, plants fine-tune the rhythmic genes to anticipate the seasonal photoperiod changes. To address the question how quinoa responds to day length changes, we investigated the characteristics of the common oscillating genes under both LD and SD in two aspects-phase and amplitude. Out of the 9131 rhythmic transcripts, only a small proportion of transcripts (855) were not phase-shifted under both conditions (Fig. 3A, Table S4). A higher percentage of transcripts were shifted by 1 h to 3 h (Fig. 3A). Comparison of the gene phase indicated that, a higher number of transcripts phased to the beginning (ZT08-ZT10) and late time (ZT18-ZT19) of dusk period under SD were shifted by LD treatment (Fig. 3B). Then we normalized the calculated phase shift value (-23 h to 23 h) to absolute value (0 h to 12 h) by adding or subtracting 24 h, and performed GO analysis to integrate the biological meaning of those phase-shifted cycling genes. GO analysis indicated that the unshifted transcripts were mainly involved in basic metabolisms such as “regulation of lipid metabolic process” and “unsaturated fatty acid biosynthetic process” (Table 2). The cycling genes shifted by 1 h to 4 h were significantly enriched in “photosynthesis, light harvesting” and “RNA methylation” (Table 2). Those transcripts shifted by 9 h to 12 h were involved in some circadian clock associated terms such as “response to absence of light” and “circadian rhythm”, and phytohormone signaling pathway like “regulation of cytokinin-activated signaling pathway” (Table 2).

**Table 2:** GO enrichment analysis of the rhythmic transcripts with phase shift and amplitude changes under SD and LD

P**hase shift**	**Number of genes**	**ID**	**Description**	**FDR**
0 h	855	GO:0,019,216	regulation of lipid metabolic process	0.004372
		GO:0,006,636	unsaturated fatty acid biosynthetic process	0.033974
		GO:0,006,334	nucleosome assembly	0.033974
		GO:0,009,611	response to wounding	0.039993
1-4 h	5733	GO:0,009,765	photosynthesis, light harvesting	0.000315
		GO:0,001,510	RNA methylation	0.009443
5-8 h	1982	-	-	-
9-12 h	561	GO:0,009,646	response to absence of light	0.021307
		GO:0,006,168	adenine salvage	0.038732
		GO:0,006,333	chromatin assembly or disassembly	0.038732
		GO:0,009,407	toxin catabolic process	0.038732
		GO:0,002,679	respiratory burst involved in defense response	0.038732
		GO:0,080,036	regulation of cytokinin-activated signaling pathway	0.038732
		GO:0,007,623	circadian rhythm	0.038732
		GO:0,050,830	defense response to Gram-positive bacterium	0.038732
		GO:0,006,817	phosphate ion transport	0.041366
		GO:0,046,506	sulfolipid biosynthetic process	0.043383
**Amplitude change**	**Number of genes**	**ID**	**Description**	**FDR**
SD-strengthened	2479	GO:0,015,996	chlorophyll catabolic process	0.021017
		GO:0,006,629	lipid metabolic process	0.02102
		GO:0,006,656	phosphatidylcholine biosynthetic process	0.021017
		GO:0,009,408	response to heat	0.021017
		GO:0,007,165	signal transduction	0.043452
		GO:0,035,556	intracellular signal transduction	0.044682
SD-attenuated	340	GO:0,000,911	cytokinesis by cell plate formation	5.57E-06
		GO:0,010,583	response to cyclopentenone	0.000188
		GO:0,008,283	cell proliferation	0.001297
		GO:0,051,225	spindle assembly	0.001801
		GO:0,043,987	histone H3-S10 phosphorylation	0.001801
		GO:0,071,277	cellular response to calcium ion	0.001801
		GO:0,051,301	cell division	0.002323
		GO:0,016,572	histone phosphorylation	0.002404
		GO:0,000,226	microtubule cytoskeleton organization	0.002941
		GO:0,031,408	oxylipin biosynthetic process	0.003735
		GO:0,009,558	embryo sac cellularization	0.005826
		GO:0,051,726	regulation of cell cycle	0.008063
		GO:0,042,127	regulation of cell proliferation	0.008169
		GO:0,009,704	de-etiolation	0.01056
		GO:0,015,994	chlorophyll metabolic process	0.01056
		GO:0,090,333	regulation of stomatal closure	0.018269
		GO:0,006,084	acetyl-CoA metabolic process	0.029092
		GO:0,000,103	sulfate assimilation	0.031358
		GO:0,010,114	response to red light	0.032921
		GO:0,000,079	regulation of cyclin-dependent protein serine/threonine kinase activity	0.039571
		GO:0,019,216	regulation of lipid metabolic process	0.046269
		GO:0,000,055	ribosomal large subunit export from nucleus	0.046269
		GO:0,019,805	quinolinate biosynthetic process	0.046269
		GO:0,042,273	ribosomal large subunit biogenesis	0.046269
		GO:0,051,176	positive regulation of sulfur metabolic process	0.046269
		GO:0,051,347	positive regulation of transferase activity	0.046269
		GO:0,070,370	cellular heat acclimation	0.046269

Moreover, we investigated the photoperiodic effect on gene expression amplitude. The amplitude of rhythmic gene was calculated by subtracting the mean value from the maximum value of the time course FPKMs. We found the expression amplitudes of 2819 cycling transcripts were significantly altered (fold change > 2.0) by different day lengths (Table 2, Table S5). Notably, majority (2479) of those genes in quinoa displayed amplitude-strengthened patterns when switched to SD (Table 2, Table S5). The SD-strengthened cycling transcripts were involved in “chlorophyll catabolic process”, “lipid metabolic process” and “signal transduction” (Table 2). By contrast, the LD-enhanced rhythmic genes (340) were participated in as many as 27 terms, which were implicated in some rhythmic events, such as “cell proliferation”, “regulation of cell cycle” and “cell division”, and light responses, such as “de-etiolation”, “chlorophyll metabolic process”, “regulation of stomatal closure” and “response to red light” (Table 2).

### The phases of most clock components were advanced by SD

The core circadian clock drives downstream rhythmic genes to sophisticatedly control various biological processes. To date, the circadian clock regulatory networks have been extensively investigated in Arabidopsis, however, the knowledge about the counterparts in quinoa remain to be explored. In this study, we used the clock genes of Arabidopsis (Table S6) as queries to BLSATP search against the proteome of quinoa (Phytozome v13.0). Then phylogenic analysis was performed to identify the putative circadian clock pathway homologs. As showed in Figure S3, most clock genes have multiple copies in quinoa genome while the *CIRCADIAN CLOCK ASSOCIATED 1* (*CCA1*) and *PSEUDO-RESPONSE REGULATOR 3* (*PRR3*) homologs were absent.

**Fig. 4:**
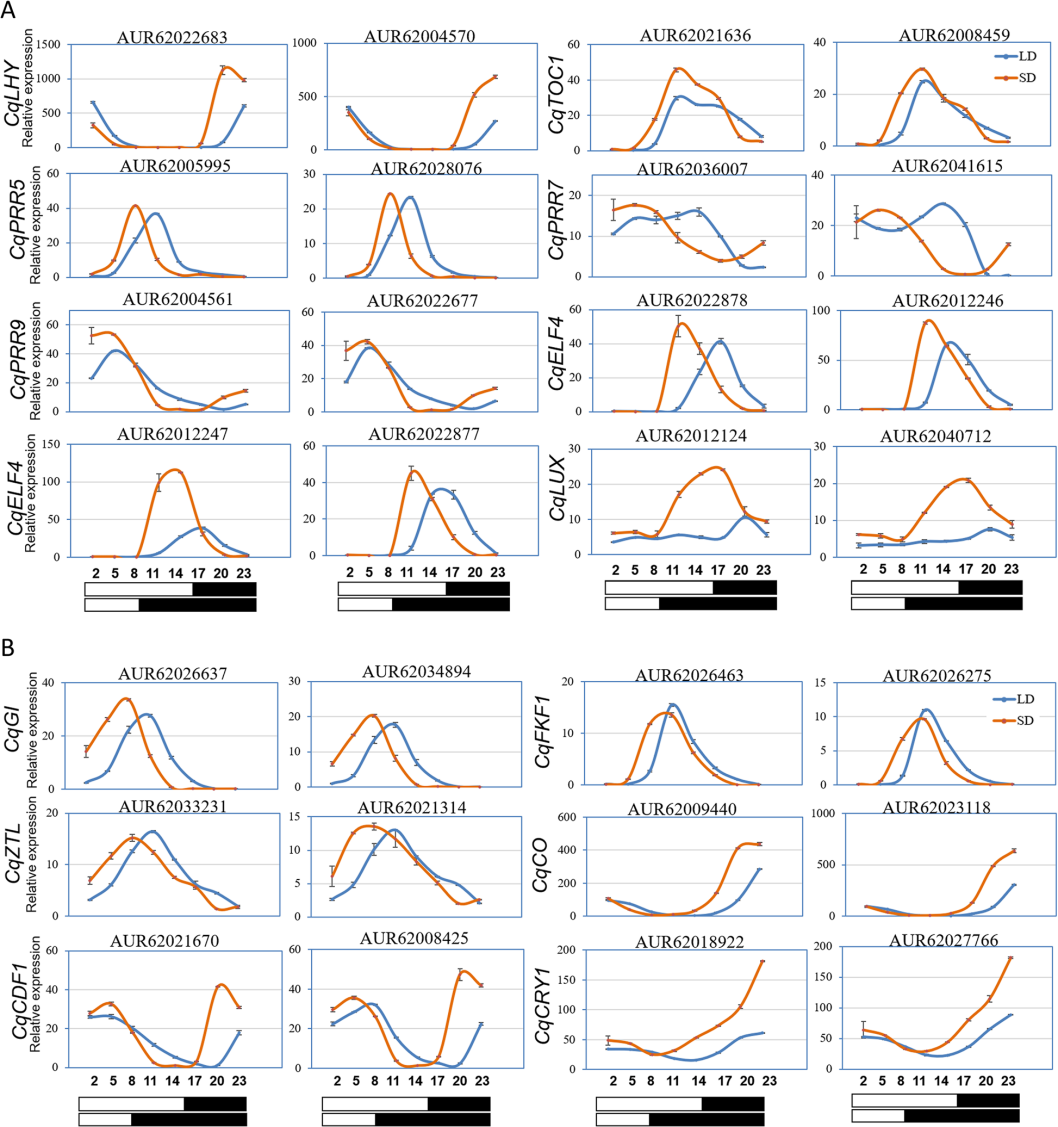
Most of the key regulators in circadian clock pathway had advanced phases and strengthened amplitudes under SD than under LD. **A** Diurnal expression analysis indicated the core circadian clock components *CqLHY*, *CqPRR5*/*7*/*9*, *CqELF4* and *CqLUX* homologs had advanced phases and strengthened amplitudes under SD. **B** The clock associated regulators *CqGI*, *CqFKF1*, *CqZTL* and the clock output gene *CqCO* were advanced, while the clock input gene *CqCRY1* and the output gene *CqCDF1* were lagged by SD

Further, the effects of photoperiod on clock gene phase were evaluated. Interestingly, the gene phase comparison results indicated that the homologous genes tended to have the same phase under the same photoperiod. For example, the homologs of *PRR5*, *EARLY FLOWERING 4* (*ELF4*-AUR62012246, AUR62022877, AUR62022878), *GIGANTEA* (*GI*), *FLAVIN-BINDING*, *KELCH REPEAT*, *F-BOX 1* (*FKF1*), *ZEITLUPE* (*ZTL*), *CONSTANS* (*CO*), *CYCLING DOF FACTOR 1* (*CDF1*) and *CRYPTOCHROME 1* (*CRY1*) were phased to ZT08, ZT12, ZT10, ZT07, ZT08, ZT22, ZT21 and ZT23 under SD (Table 3, Fig. 4), respectively. Under LD, the *LHY*, *PRR7*, *PRR9*, *LUX ARRYTHMO* (*LUX*), *GI*, *FKF1*, *CqZTL*, *CO* and *CDF1* homologs were phased to ZT01, ZT09, ZT06, ZT20, ZT10, ZT12, ZT11, ZT23 and ZT04 (Table 3, Fig. 4), respectively. We noticed that the *ELF3* homologs (AUR62043053, AUR62040202) displayed arrhythmic transcription when switched to LD. Besides, most (20 out of 28) of those clock related components had advanced phases when switched from LD to SD (Table 3, Fig. 4).

**Table 3:** The phase shifts and amplitude changes of circadian clock associated homologs under SD and LD

Gene name	ID	Phase-SD (ZT)	Phase-LD (ZT)	Phase shift	Amplitude ratio (SD/LD)
*CqLHY*	AUR62004570	00	01	-1 h	1.65
*CqLHY*	AUR62022683	20	01	19 h	1.73
*CqTOC1*/*PRR1*	AUR62008459	12	11	1 h	1.15
*CqTOC1*/*PRR1*	AUR62021636	13	12	1 h	1.76
*CqPRR5*	AUR62005995	08	10	-2 h	1.21
*CqPRR5*	AUR62028076	08	11	-3 h	1.12
*CqPRR7*	AUR62036007	05	09	-4 h	1.35
*CqPRR7*	AUR62041615	04	09	-5 h	1.11
*CqPRR9*	AUR62004561	03	06	-3 h	1.27
*CqPRR9*	AUR62022677	06	06	0 h	1.08
*CqELF4*	AUR62012246	12	15	-3 h	1.34
*CqELF4*	AUR62012247	13	16	-3 h	2.98
*CqELF4*	AUR62022877	12	16	-4 h	1.47
*CqELF4*	AUR62022878	12	17	-5 h	1.22
*CqLUX*	AUR62012124	15	20	-5 h	2.20
*CqLUX*	AUR62040712	16	20	-4 h	3.15
*CqGI*	AUR62026637	07	10	-3 h	1.24
*CqGI*	AUR62034894	07	10	-3 h	1.15
*CqFKF1*	AUR62026275	10	12	-2 h	0.85
*CqFKF1*	AUR62026463	10	12	-2 h	0.79
*CqZTL1*	AUR62021314	08	11	-3 h	0.90
*CqZTL1*	AUR62033231	08	11	-3 h	0.85
*CqCO*	AUR62009440	22	23	-1 h	1.38
*CqCO*	AUR62023118	22	23	-1 h	2.00
*CqCDF1*	AUR62008425	21	04	17 h	1.50
*CqCDF1*	AUR62021670	21	04	17 h	1.76
*CqCRY1*	AUR62018922	23	21	2 h	4.11
*CqCRY1*	AUR62027766	23	00	23 h	2.53

As to the core clock, the dawn-phased *CqLHY* were shifted to early morning when switched to LD (Fig. 4A). The evening expressed *TIMING OF CAB EXPRESSION 1* (*CqTOC1*, namely *CqPRR1*) and *CqPRR5* homologs were phase-shifted to light rather than darkness as the day length increased (Fig. 4A). The morning-phased *CqPRR7*, *CqPRR9* and the evening-phased *CqELF4* (AUR62012247, AUR62022877, AUR62022878) and *CqLUX* had obvious lagged phases as daytime lengthened, but remained peaked in light and dark periods, respectively (Fig. 4A). As to the additional clock feedback loops, similar to *CqTOC1*, the evening-phased *CqGI*, *CqFKF1* and *CqZTL* had delayed phases when switched from SD to LD (Fig. 4B), thus having longer duration of light exposure. Next, we detected the expression of clock input and output genes. The evening-phased *CqCDF1* had lagged phases and exposed to light when switched from SD to LD (Fig. 4B). The phases of *CqCRY1* and *CqCO* were just slightly shifted and peaking in dark time (Fig. 4B).

Moreover, we investigated the amplitude changes of those clock genes under different photoperiods. The amplitude changes of *CqPRR5*, *CqPRR7*, *CqPRR9*, *CqELF4* (AUR62012246, AUR62022877, AUR62022878), *CqGI*, *CqFKF1* and *CqZTL* homologs were not significant (less than 1.5 folds), in which only *CqFKF1* and *CqZTL* were weakened by SD (Table 3, Fig. 4). By contrast, *CqLHY*, *CqTOC1* (AUR62021636), *CqELF4* (AUR62012247), *CqLUX*, *CqCO* (AUR62023118), *CqCRY1* had significantly strengthened amplitudes (larger than 2 folds) when switched to SD (Table 3, Fig. 4). Collectively, the phase and amplitude changes in combination with the duration of light exposure under different photoperiods may further influence the transcriptional rhythm of downstream genes, and the overall outcoming effects on quinoa growth and development were worth investigating.

### Transcriptional changes of transcription factors under LD and SD

**Fig. 5:**
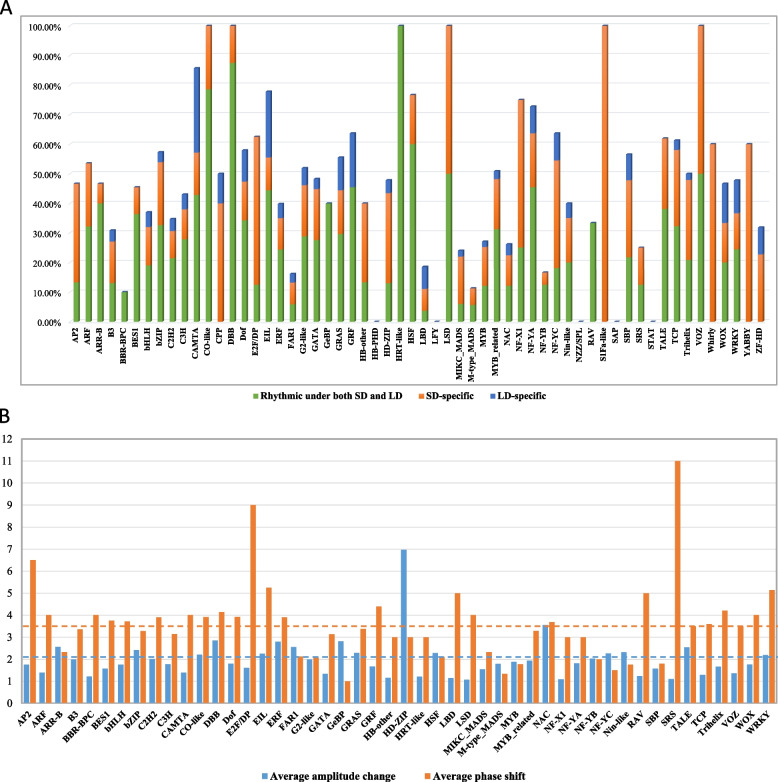
Identification of the diurnal rhythmic transcription factors and expression changes under SD and LD. **A** Percentage of the commonly rhythmic, SD- and LD-specific rhythmic transcription factors in each family. **B** Comparison of the average phase shift and amplitude change values of in each transcription factors family between under SD and under LD. Only the commonly oscillating transcription factors under both photoperiods were used for analysis

Transcription factors (TFs) play pivotal roles in gene expression regulation and participate in various biological events. To what extent the photoperiod influences TF expression remains yet to be uncovered in quinoa. In this study, we identified the global TFs and investigated their expression changes under different day lengths. Out of 2093 TFs in quinoa genome, 40.56% (849) TFs displayed rhythmic expression patterns under at least one condition, comparable to the percentage of global rhythmic genes (Table 4). 35 families showed overrepresentation in diurnal rhythmic expression, with higher percentages than 40.56% (Table 4, Fig. 5A). The SD-induced rhythmic TFs were more abundant than LD-induced (Table 4, Fig. 5A). 444 TFs (21.21%) from 48 families were diurnal cycling under both conditions (Table 4, Fig. 5A). Among all the TF families, 25 families harbored rhythmic TFs with percentage larger than 50% (Table 4, Fig. 5A). Remarkably, the percentages of rhythmic TFs in CO-like, DBB, HRT-like, LSD, S1Fa-like and VOZ families reached 100% (Table 4, Fig. 5A). We found that the diurnal rhythms of S1Fa-like, Whirly and YABBY were SD-specific, while the TFs in BBR-BPC, GeBP, HRT-like and RAV families remained diurnal rhythmic under both conditions (Table 4, Fig. 5A).

**Table 4:** Statistic of the rhythmic transcription factors under different photoperiods

TF family	Total TFs	Rhythmic under at least one condition	Percentage	Rhythmic under both conditions	Percentage	SD-specific	Percentage	LD-specific	Percentage
AP2	15	7	46.67%	2	13.33%	5	33.33%	0	0.00%
ARF	28	15	53.57%	9	32.14%	6	21.43%	0	0.00%
ARR-B	15	7	46.67%	6	40.00%	1	6.67%	0	0.00%
B3	107	33	30.84%	14	13.08%	15	14.02%	4	3.74%
BBR-BPC	10	1	10.00%	1	10.00%	0	0.00%	0	0.00%
BES1	11	5	45.45%	4	36.36%	1	9.09%	0	0.00%
bHLH	200	74	37.00%	38	19.00%	26	13.00%	10	5.00%
bZIP	89	51	57.30%	29	32.58%	19	21.35%	3	3.37%
C2H2	98	34	34.69%	21	21.43%	9	9.18%	4	4.08%
C3H	79	34	43.04%	22	27.85%	8	10.13%	4	5.06%
CAMTA	7	6	85.71%	3	42.86%	1	14.29%	2	28.57%
CO-like	14	14	100.00%	11	78.57%	3	21.43%	0	0.00%
CPP	10	5	50.00%	0	0.00%	4	40.00%	1	10.00%
DBB	8	8	100.00%	7	87.50%	1	12.50%	0	0.00%
Dof	38	22	57.89%	13	34.21%	5	13.16%	4	10.53%
E2F/DP	8	5	62.50%	1	12.50%	4	50.00%	0	0.00%
EIL	9	7	77.78%	4	44.44%	1	11.11%	2	22.22%
ERF	123	49	39.84%	30	24.39%	13	10.57%	6	4.88%
FAR1	136	22	16.18%	8	5.88%	10	7.35%	4	2.94%
G2-like	52	27	51.92%	15	28.85%	9	17.31%	3	5.77%
GATA	29	14	48.28%	8	27.59%	5	17.24%	1	3.45%
GeBP	5	2	40.00%	2	40.00%	0	0.00%	0	0.00%
GRAS	54	30	55.56%	16	29.63%	8	14.81%	6	11.11%
GRF	11	7	63.64%	5	45.45%	0	0.00%	2	18.18%
HB-other	15	6	40.00%	2	13.33%	4	26.67%	0	0.00%
HB-PHD	1	0	0.00%	0	0.00%	0	0.00%	0	0.00%
HD-ZIP	46	22	47.83%	6	13.04%	14	30.43%	2	4.35%
HRT-like	2	2	100.00%	2	100.00%	0	0.00%	0	0.00%
HSF	30	23	76.67%	18	60.00%	5	16.67%	0	0.00%
LBD	54	10	18.52%	2	3.70%	4	7.41%	4	7.41%
LFY	3	0	0.00%	0	0.00%	0	0.00%	0	0.00%
LSD	2	2	100.00%	1	50.00%	1	50.00%	0	0.00%
MIKC_MADS	50	12	24.00%	3	6.00%	8	16.00%	1	2.00%
M-type_MADS	53	6	11.32%	3	5.66%	3	5.66%	0	0.00%
MYB	107	29	27.10%	13	12.15%	14	13.08%	2	1.87%
MYB_related	112	57	50.89%	35	31.25%	19	16.96%	3	2.68%
NAC	107	28	26.17%	13	12.15%	11	10.28%	4	3.74%
NF-X1	4	3	75.00%	1	25.00%	2	50.00%	0	0.00%
NF-YA	11	8	72.73%	5	45.45%	2	18.18%	1	9.09%
NF-YB	24	4	16.67%	3	12.50%	1	4.17%	0	0.00%
NF-YC	11	7	63.64%	2	18.18%	4	36.36%	1	9.09%
Nin-like	20	8	40.00%	4	20.00%	3	15.00%	1	5.00%
NZZ/SPL	1	0	0.00%	0	0.00%	0	0.00%	0	0.00%
RAV	3	1	33.33%	1	33.33%	0	0.00%	0	0.00%
S1Fa-like	1	1	100.00%	0	0.00%	1	100.00%	0	0.00%
SAP	2	0	0.00%	0	0.00%	0	0.00%	0	0.00%
SBP	23	13	56.52%	5	21.74%	6	26.09%	2	8.70%
SRS	8	2	25.00%	1	12.50%	1	12.50%	0	0.00%
STAT	1	0	0.00%	0	0.00%	0	0.00%	0	0.00%
TALE	21	13	61.90%	8	38.10%	5	23.81%	0	0.00%
TCP	31	19	61.29%	10	32.26%	8	25.81%	1	3.23%
Trihelix	48	24	50.00%	10	20.83%	13	27.08%	1	2.08%
VOZ	4	4	100.00%	2	50.00%	2	50.00%	0	0.00%
Whirly	5	3	60.00%	0	0.00%	3	60.00%	0	0.00%
WOX	15	7	46.67%	3	20.00%	2	13.33%	2	13.33%
WRKY	90	43	47.78%	22	24.44%	11	12.22%	10	11.11%
YABBY	10	6	60.00%	0	0.00%	6	60.00%	0	0.00%
ZF-HD	22	7	31.82%	0	0.00%	5	22.73%	2	9.09%

As to the cycling TFs under both conditions, we investigated their amplitude changes and phase shifts in response to different day lengths. The average amplitude changes and phase shifts of all rhythmic TFs were 2.14 folds and 3.43 h, respectively (Fig. 5B). The amplitude changes of 16 TF families exceeded the overall mean value (Fig. 5B). HD-ZIP family had the largest average amplitude change, reaching 6.97 folds (Fig. 5B). There were 25 TF families whose average phase shifts were larger than the overall mean value (Fig. 5B). SRS family was subjected to the largest phase shift when treated by to different photoperiods (Fig. 5B). Remarkably, both the amplitude changes and phase shift values of several TF families, including CO-like, DBB, EIL, ERF, NAC, TALE and WRKY, were larger than the mean values (Fig. 5B), respectively, indicating those TFs are sensitive to the day length changes and may function as key mediators for output of the core circadian clock of quinoa.

### Mining of time-of-day specific motifs in the rhythmic gene promoters

Diurnal oscillating transcription depends on the specific interaction between TFs and the *cis* elements. We used STREME [27] to discover the enriched motifs in the promoters of different bins under different conditions. SEA [28] was adopted to calculate the frequency of each motif in different bins and entire rhythmic genes under SD and LD, respectively. The Z-score curve of each motif at different time points was used to evaluate the time-of-day specificity. The studies in Arabidopsis showed that the circadian clock regulates downstream cycling genes by binding to several overrepresented circadian clock regulatory elements (CCREs) including AtCCA1‐ and AtLHY‐binding sites (CBS, AAAAAATCT), G‐box‐like motif (CACGTG), GATA box (GATA), morning element (ME, CCACAC), evening element (EE, AATATCT), telomere box (TBX, AAACCCT), protein box element (PBX, ATGGCC) and starch box (SBX, AAGCCC). Among those elements, we found only G‐box‐like motif and TBX showed significantly time-of-day enrichment in quinoa rhythmic gene promoters (Figure S4). The G‐box‐like motif was specifically enriched in the promoters of cycling transcripts phased to the light period under SD but not under LD (Figure S4). TBX displayed time-of-day specific enrichment in the promoters of cycling transcript phased to the end of dark period regardless of day length change (Figure S4). However, the other CCREs were not found to be enriched in the rhythmic gene promoters under neither condition. These results suggest the partial conserved *cis*-regulatory modules between Arabidopsis and quinoa.

**Fig. 6:**
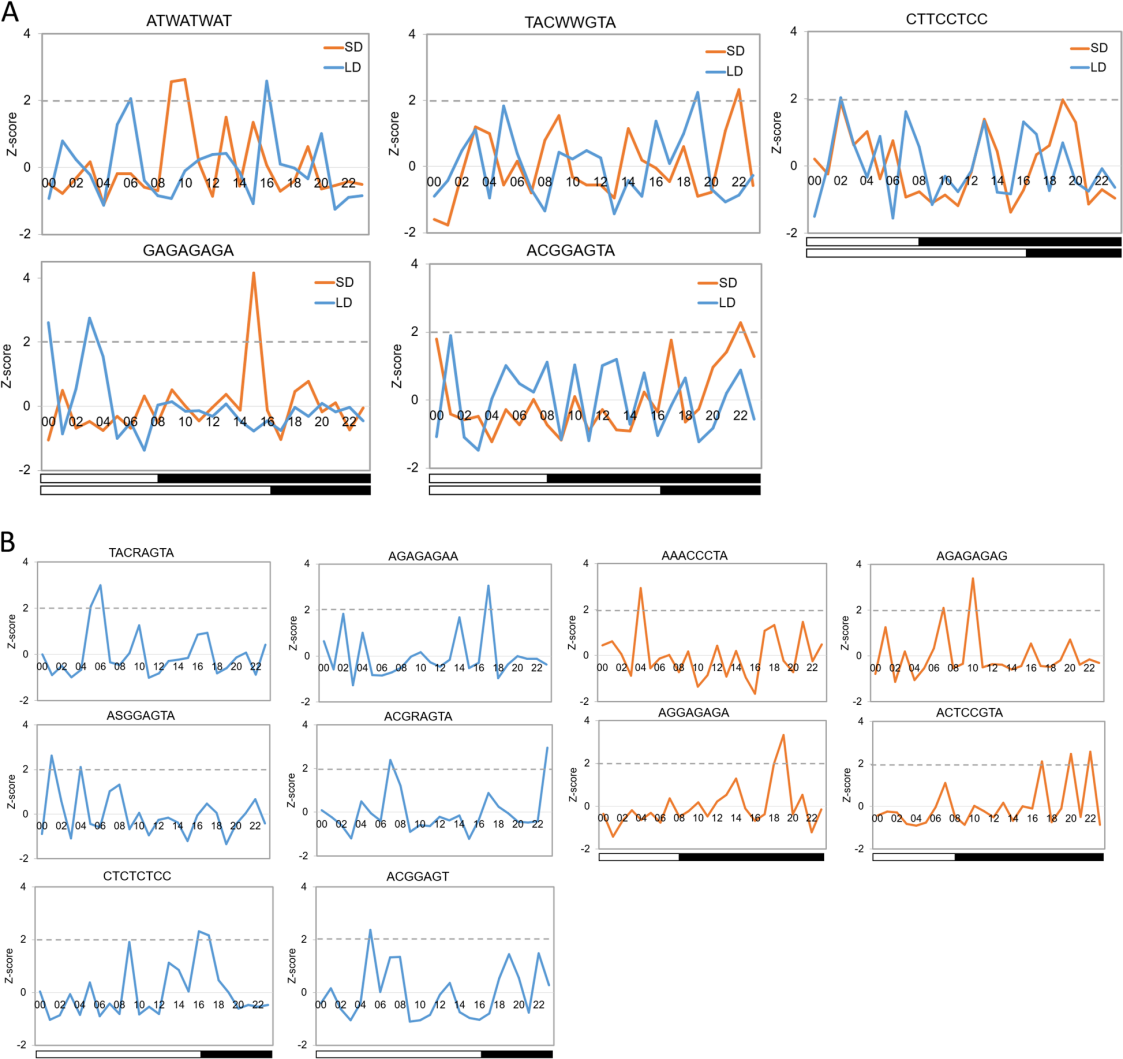
Frequencies of the *cis* elements with time-of-day specific enrichment. **A** Z-score profiles of 5 *cis* elements with time-of-day specific enrichment under both conditions. **B** Z-score profiles of 6 and 4 *cis* elements with time-of-day specific enrichment under LD and SD, respectively

In addition, we identified 5 novel putative *cis* elements with specific enrichment under both conditions. The motif “ATWATWAT (W = A or T)” was specifically enriched in the promotors of transcripts phased to evening (ZT09 to ZT10) under SD whereas was enriched in the promotors of transcripts phased to ZT06 and ZT16 under LD (Fig. 6A). The motif “TACWWGTA (W = A/T)” had higher Z-score at ZT22 and ZT19 under SD and LD (Fig. 6A), respectively. “CTTCCTCC” and “GAGAGAGA” motifs were more abundant in the dark period under SD, while was phased to light period under LD (Fig. 6A). The motif “ACGGAGTA” was significantly enriched at late dark time under SD (Fig. 6A). Besides, we also noticed that 6 and 4 motifs were LD- and SD-specific, respectively. The “TACRAGTA (R = A/G)”, “ASGGAGTA (S = C/G)”, “ACGRAGTA” and “ACGGAGT” motifs were specifically distributed in cycling genes phased to the light period under LD (Fig. 6B). The “AGAGAGAA” and “CTCTCTCC” motifs were significantly phased to the evening time under LD (Fig. 6B). Under SD, the “AAACCCTA” motif was highly enriched in bins of light period (Fig. 6B). The “AGAGAGAG” motif was phased to the evening time, and the “AGGAGAGA” and “ACTCCGTA” motifs were overrepresented at the late dark time (Fig. 6B). Then we submitted those overrepresented motifs to JASPAR NONREDUNDANT DNA-JASPAR CORE (2018) plants database [29] to find whether they matched with the known transcription factor binding sites (TFBS). Finally, we found only 3 motifs were matched with the known TFBS. The alignment results showed that the motifs “GAGAGAGA” and “AGAGAGAG” highly matched with RAMOSA1 (Table 5), which is a known TFBS of C2H2 zinc finger factors. The motif “AAACCCTA” was highly similar to the sequence of AT1G72740 that is a TFBS of Helix-Turn-Helix MYB-related proteins (Table 5). Collectively, those obtained motifs may be novel *cis* elements involved in rhythm controlling in quinoa.

**Table 5:** Comparison results of the overrepresented motifs with known TFBS in JASPAR CORE (2018) database

Motif sequence	Target ID	E-value	Target name	Class	Optimal alignment
GAGAGAGA	MA1416.1	0.006	RAMOSA1	C2H2 zinc finger factors	
AAACCCTA	MA1353.1	0.008	AT1G72740	Helix-Turn-Helix (MYB-related)	
AGAGAGAG	MA1416.1	0.006	RAMOSA1	C2H2 zinc finger factors	

## Discussion

Accumulating evidences have displayed the importance of clock genes for local adaption as well as yield improvement in crops. The decelerated circadian clock is for highly associated with the pronounced performance of domesticated tomato (*Solanum lycopersicum*) cultivars [10]. Mutation of *CCA1* by RNA interference (RNAi) increased chlorophyll and starch content in Arabidopsis [30]. Thus, deciphering the circadian clock architecture and the rhythm-keeping mechanisms is the first step towards understanding and ultimately improving the yield of quinoa. Fortunately, as a model plant in Amarantheae, opening of the high-quality genome information has provided an opportunity for molecular design breeding in future [19].

Photoperiod is a major factor shaping the phase and amplitude of clock associated genes. A series of evidences have demonstrated the central roles of photoperiod in controlling quinoa growth, flowering, maturation and yield [16, 31, 32]. Seed size and leaf number were affected when applied with different photoperiods [16]. Longer photoperiod inhibited floral transition in most quinoa germplasms [31]. When switched from SD to LD, days to flowering and maturity were prolonged, and plant height and nodes number were increased [32]. These evidences indicate that quinoa is a photoperiod-sensitive plant, yet, the linkage between internal global rhythmic genes expression and those photoperiodic reactions has not been fully understood. Until recently, a study covering 303 quinoa accessions indicated that the delayed flowering time of SD type quinoa accessions was highly associated with 5 genotypes of *CqFT* and *CqCOL* [17]. In addition, our group found that 1 h night break in the middle of dark period under SD significantly repressed floral transition in quinoa, which may result from the regulatory effects of several lncRNAs on the expression of *CqFT*, *CqCO* and some photoreceptors and clock components [18]. Those studies only investigated the photoperiod effects on several key genes at single or a few time points, the full picture of gene expression changes remains to be uncovered. In this study, we performed global transcriptional level analysis of quinoa leaves sampled through diurnal cycles of 24 h and compared the full-time diurnal expression variations under SD and LD. Using HAYSTACK algorithm, We found as many as 44% of global transcripts in quinoa were diurnal rhythmic under at least one photoperiod (Fig. 1), larger than the proportion of periodic genes in *Brachypodium distachyon* (19.5%) [33], poplar (30.3%) and rice (40.8%) [24], while less than that in the shoots (76.54%) and roots (50.97%) of *Medicago truncatula* [34]. We found SD obviously rendered more genes rhythms than LD (Fig. 1). Meanwhile, a lot of transcripts were SD- and LD-specific cycling. These results were in consistence with the observations in Arabidopsis [22], indicating the rhythms of many genes are dependent on special diurnal conditions. GO analysis indicated that the rhythmic genes phased to different time points were participated in time-of-day specific biological processes. Comparison of SD and LD items indicated that the majority of GO items for LD phase bins were not appeared in SD phase bins, and the item “metabolic process” was phase-shifted from ZT1-ZT3 to ZT7-ZT9 when switched to SD (Fig. 2). These differences in biological processes caused by photoperiod may further lead to growth and developmental variations. The phase topology map between SD and LD showed that a higher percentage of transcripts phased to the middle and end of photoperiod under LD were advanced by 2-3 h under SD (Fig. 3B), which is similar to the findings in Arabidopsis [22], indicating governing role of diurnal condition on rhythm regulation over differing species. As to the common oscillating transcripts under both photoperiods, we found only a small proportion (9.36%) was not phase-shifted (Fig. 3). It is unsurprising that the shifted genes were categorized in “photosynthesis, light harvesting”, “circadian rhythm”, because external light condition interacts with circadian clock to control the overall rhythms (Table 2). We found the amplitudes of about 30% of the common oscillating genes were significantly affected by photoperiod (Table 2). The majority were strengthened by SD (Table 2), in consistence with the strengthened expressions of core clock components (Fig. 4). Those amplitude-strengthened or -attenuated cycling genes were associated with abiotic response, light response and cell cycling responses (Table 2), indicating quinoa may reshape those processes to improve adaption to photoperiod changes.

This study revealed the putative circadian clock architecture and assessed their expression patterns in different photoperiods. As quinoa is an allotetraploid plant, multiple copies were identified for all clock homologs. The similarity in expression patterns between copies may suggest their functional redundancies. Comparison of the phases of the core clock components in different plants showed that, under LD, *CqLHY* and *CqTOC1* peaked around ZT01 and ZT12 (Fig. 4), respectively, consistent with their expression patterns in Arabidopsis, rice, lettuce (*Lactuca sativa*) [24, 35]. *CqFKF1* peak around ZT12 (Fig. 4), similar with rice while differing from Arabidopsis [24]. *CqGI* peaked around ZT10 (Fig. 4), differing from either species. Compared with the *LUX* homologs of Arabidopsis, rice and poplar peaking at dusk time [24], in quinoa *CqLUX* had distinct expression patterns which peaked around the middle of dark period (Fig. 4). *ELF3* (AUR62043053, AUR62040202) under LD and *PHYA* homologs (AUR62003557, AUR62017871) under SD were not found to be rhythmic, respectively, consistent with the expression pattern in lettuce or tomato [35, 36]. Those observations suggested that to some extent the diurnal expression patterns of core clock components in quinoa are partially conserved with other plants although it needs further validation by comparing expression patterns under exactly the same light intensity and photoperiod. The phase shift of clock regulators combined with light length changes usually leads to expression variation of output genes. This has been clearly evidenced by the GI-FKF1 protein complex regulatory roles on *CO* and *FT* [7]. Coincidently, we found that, in consistence with *CqGI* and *CqFKF1* homologs expression trends, the output gene *CqCO* were advanced and strengthened by SD (Fig. 4), and consequently two downstream flowering integrator *CqFT-LIKE* homologs (AUR62006619, AUR62026237) were more abundant (Figure S5). This result may partially explain the advanced flowering time by SD in quinoa. Meanwhile, we also performed real-time PCR to validate the diurnal expression patterns of several genes (*CqTOC1*, *CqLUX*, *CqELF3*, *CqGI*, *CqCO* and *CqFT-LIKE*) under different photoperiods (Figure S6). We found high consistency between RNA-seq data and real-time PCR results (Fig. 4, Figure S6), indicating the RNA-seq analysis results are highly reliable.

Rhythmic transcription relies on the specific binding between TFs and *cis* elements in gene promotor. We identified 40.56% (848) of global TFs were cycling in quinoa (Table 4, Fig. 5A), comparable to the rhythmic TFs percentage in pineapple. 25 TF families harbored a high fraction (> 50%) of rhythmic TFs (Fig. 5A). The important TFs including CO-like, DBB, HRT-like, LSD, S1Fa-like and VOZ families were 100% rhythmic (Table 4, Fig. 5A). Interestingly, we found that 3 TF families (S1Fa-like, Whirly and YABBY) were SD-specific while 0 TF family was LD-specific (Fig. 4). Some TFs involved in flowering, abiotic stress and developmental regulation, such as CO-like, DBB, EIL, ERF, NAC, TALE and WRKY, displayed hypersensitive responses when treated by differing light lengths (Fig. 5). We speculated that those TFs may function as key mediators for adaption to different photoperiods. Global survey of the motifs in different time phase bins indicated that *cis* elements G‐box‐like and TBX (Figure S4) showed similar time-of-day distribution as that in Arabidopsis, rice and poplar [24]. Besides, we found 15 novel potential *cis* elements that may be involved in rhythmic controlling in quinoa (Fig. 6).

## Conclusions

To sum up, this study identified the core clock architecture, key output TFs and *cis* elements that are important for diurnal rhythm regulation and may be potential targets for yield improvement in future. The obtained results help deepen our understanding of photoperiodic responses in quinoa, and provide a reference for studying other crops in Amaranthaceae.

## Methods

### Growth conditions and diurnal sampling of quinoa leaves

Quinoa (*Chenopodium quinoa*) cultivar “HL1” was used for diurnal sampling in this study. “HL1” is a short-day type accession sensitive to night-break treatment (NB) and displayed delayed flowering phenotype when treated by NB [37]. Quinoa seeds were sown in four soil pots and grown in controlled growth chamber (Jiang Nan, Ning Bo, China). After emergence, spared seedlings were diminished and only maintain the robust plants. Seedlings were exposed to a photoperiod of 14 h light/ 10 h dark for 7 d. Then these seedlings were transferred to long-day conditions (LD) consisting of 16 h light and 8 h dark. After entrainment in LD for 7 d, we began to collect the leaf samples of two pots for LD. For each time point, the top two-fully-expanded leaves of 3 ~ 5 plants were harvested. The leaf samples with two biological replicates were collected at Zeitgeber Time 02 (ZT02), ZT05, ZT08, ZT11, ZT14, ZT17, ZT20 and ZT23. The collected samples were immediately frozen by liquid nitrogen and stored in -80℃ fridge before RNA extraction. Afterwards, the growth chamber was adjusted to short-day conditions (SD) with relative light/dark length of 8 h/ 16 h. Three days later, leaf samples of the other two pot plants were collected under SD at the same time points as in LD. All the leaf samples were collected at about 10 ~ 14 d before bolting. At this stage the plants harbored about 4–5 true leaves. The day- and dark-time temperatures were set at 25℃ and 23℃, respectively, with the humidity of 70% and light intensity of 500 μmol^.^m^−2.^s^−1^ for all conditions.

### High-throughput sequencing and data analysis

Total RNA extraction and RNA-seq were conducted as previously reported [37, 38]. RNA samples with RIN (RNA integrity number) value larger than 7.5 were used for cDNA library construction and high-throughput sequencing on an Illumina platform (Hiseq 4000). The produced pair-end 150 bp (PE150) reads were filtered to obtain clean data. By using HISAT2 [20], clean reads were mapped to the quinoa reference genome [19]. StringTie [21] was used to quantify the relative gene expression levels by calculating the fragments per kilo base of transcript per million fragments mapped (FPKM) value. Based on the FPKM values, the Pearson's Correlation Coefficient (PCC) between samples were calculated. The result showed that all gene expression levels between biological replicates were highly correlated (R2 > 0.984) (Table S7).

### Selection of diurnal cycling transcripts

The gene mean FPKM value at different time points were calculated to obtain the LD and SD expression profiles. Further, based on the 636 models (Table S1) derived from previous studies [23, 25], diurnal rhythmic transcripts were identified by using HAYSTACK tool (http://haystack.mocklerlab.org) [22]. The parameters of HAYSTACK tool were set as: correlation cutoff ≥ 0.8, largest value dividing smallest value ≥ 2; *p*-value ≤ 0.01; background cutoff: FPKM ≥ 1. Thus, the phase and best-fit model of diurnal cycling genes under LD and SD were determined. The amplitude of rhythmic gene was measured by subtracting the mean value from the maximum value among 8 time points.

### Identification of circadian clock homologs in quinoa

The circadian clock related homologs were identified using BLASTP program. The protein sequences of circadian clock components in *Arabidopsis thaliana* were input as queries to search against the quinoa proteome (Phytozome v13.0). The best matches with cutoff *E*-value of 1e-30 were then subjected to phylogenic analysis by using MEGA v11.0. The protein sequences of Arabidopsis, rice and quinoa were aligned by using ClustalW. The phylogenic tree was constructed using the Neighbor-Joining method with the bootstrap value of 1000 replicates. The genes clustered into the same clade with Arabidopsis and rice sequences were identified as the homologs of quinoa. The circadian clock gene identifiers of Arabidopsis and rice were listed in Table S6.

### Time-of-day specific motif enrichment analysis

Based on the HAYSTACK analysis results, the LD and SD rhythmic genes were selected and categorized into 24 (ZT00 to ZT23) phase bins, respectively. By using TBtools software the 1 k bp promoter upstream sequences of genes from different phase bins were extracted. The promoter sequences of the same phase bin were further subjected to Sensitive, Thorough, Rapid, Enriched Motif Elicitation analysis (STREME) [27] to find the enriched motifs (motif width = 3–8-mer, *p*-value ≤ 0.01). Then, all the significantly enriched motifs of 24 phase bins of LD and SD were piled together, respectively, to select the non-redundant motifs. The frequencies of enriched motifs were calculated for different phase bins and all rhythmic genes, respectively, by applying Simple Enrichment Analysis (SEA) [28]. Next, the frequencies of each motif at different time points were normalized by dividing the frequencies for all rhythmic genes of LD and SD, respectively. Then the Z-score value of each motif at different time points was obtained by using STANDARDIZE analysis in Excel 2016 (Microsoft, Washington, USA). To know whether the overrepresented motifs match with the known transcription factor binding sites (TFBS) in plants, the motif sequences were submitted to motif comparison tool TOMTOM to find the best matched TFBS in “JASPAR NONREDUNDANT DNA-JASPAR CORE (2018) plants” database [29] with E-value threshold of 0.01.

### Transcription factor prediction

The protein sequences of target genes were downloaded from Phytozome v13.0, and then were submitted to PlantTFDB v5.0 to predict transcription factors of different families as previously described [39].

### Gene ontology analysis

Singular Enrichment Analysis tool in AgriGO v2.0 [26] was used for Gene ontology (GO) analysis. GO terms with False Discovery Rate (FDR) ≤ 0.05 were identified as significantly enriched terms.

### Real-time PCR

Five micrograms of total RNA were used for DNA digestion and reverse transcription to synthesize first-strand cDNA using EasyScript One-Step gDNA Removal and cDNA Synthesis SuperMix Kit (Transgen Biotech, Beijing, China). Then, the diluted cDNA was used to perform real-time PCR on a real-time PCR system (qTOWER3, Analytik Jena, Germany). The real-time PCR program was set as: 95 °C for 2 min, 39 cycles of denaturing at 95 °C for 5 s, annealing and extension at 58 °C for 15 s. *CqUBQ10* (ID: AUR62015654) was used as internal control gene. The relative expression levels were obtained by using 2^−△△Ct^ algorithm from three replicates. The primers used for real-time PCR were list in Table S8.

## Supplementary Information

**Additional file 1: Figure S1.** Phenotypes of quinoa plants grown under SD and LD at 60 days after sowing. The “HL1” plants grown under SD had advanced flowering time than plants grown under LD. **Figure S2.** Examples of various models shifted to ZT08. The models used to identify diurnal rhythmic transcripts include Asymt1, Asymt2, Box1, Box1.5, Box2, Cos, hBox, Mt, Rigid and Spike. **Figure S3.** Phylogenic analysis of the circadian clock homologs between Arabidopsis, rice and quinoa. Proteins of (A)LHY-CCA, (B) PRR, (C) ELF3, (D) ELF4, (E) LUX, (F) GI, (G) FKF1-ZTL, (H) CO, (I) CDF and (J) CRY families of Arabidopsis, rice and quinoa were used to construct phylogenic tree using the Neighbor-Joining method. The bootstrap value was 1000 replicates. **Figure S4.** Frequencies of the representative CCREs under different photoperiods. Z-score profiles of G‐box (CACGTG) and TBX (AAACCCT) in the promoters of rhythmic transcripts of different phase bins under SD and LD were calculated. **Figure S5.** Expressions of two *CqFT-LIKE* homologs under SD and LD. mRNA abundances of *CqFT-LIKE* genes in quinoa leaves under SD were much higher than that under LD. **Figure S6.** Real-time PCR validation of gene diurnal expression patterns under SD and LD. *CqTOC1*, *CqLUX*, *CqELF3*, *CqGI*, *CqCO* and *CqFT-LIKE* were selected for real-time PCR test. The orange and green curves stand for relative gene expression levels under SD and LD, respectively. The real-time PCR values are mean ± SD (*n* = 3).

**Additional file 2: Table S1.** 636 HAYSTACK models used to determine diurnal rhythmicity. **Table S2. **Rhythmic genes under LD. **Table S3. **Rhythmic genes under SD. **Table S4. **The phase shift of 9131 commonly rhythmic genes under SD and LD. **Table S5. **The amplitude changes of 9131 commonly rhythmic genes under SD and LD. **Table S6.** List of circadian clock pathway genes in Arabidopsis and rice. **Table S7.** Pearson's Correlation Coefficient between samples. **Table S8.** Primers used for real-time PCR analysis.

## Data Availability

The RNA-seq data generated from the diurnally collected quinoa leaf samples in this study are deposited at NCBI SRA database (http://trace.ncbi.nlm.nih.gov/Traces/sra) under BioProject accessions of PRJNA824606, PRJNA825321, PRJNA824547, PRJNA824641, PRJNA824640, PRJNA824668, PRJNA824959, PRJNA824960, PRJNA824961, PRJNA824962 and PRJNA824963.

## Abbreviations

SD: Short-day
LD: Long-day conditions
NB: Night-break
CCA1: CIRCADIAN CLOCK ASSOCIATED 1
LHY: LATE ELONGATED HYPOCOTYL
PRR1: PSEUDO RESPONSE REGULATOR 1
TOC1: TIMING OF CAB EXPRESSION1
GI: GIGANTEA
FKF1: FLAVIN-BINDING, KELCH REPEAT, F-BOX 1
PHY: PHOTOCHROME
CRY: CRYPTOCHROME
CO: CONSTANS
CDF: CYCLING DOF FACTOR
FT: FLOWERING LOCUS T
PIF: PHYTOCHROME INTERACTING FACTOR
CBF: C-REPEAT BINDING FACTOR
ELF: EARLY FLOWERING
CCREs: Circadian clock regulatory elements
CBS: AtCCA1- and AtLHY-binding sites
ME: Morning element
EE: Evening element
TBX: Telomere box
PBX: Protein box element
SBX: Starch box
RNAi: RNA interference
FPKM: Fragments Per Kilobase of transcript sequence per Millions base pairs sequenced
GO: Gene ontology
KEGG: Kyoto Encyclopedia of Genes and Genomes
STREME: Sensitive, Thorough, Rapid, Enriched Motif Elicitation analysis
SEA: Simple Enrichment Analysis
TFBS: Transcription factor binding sites
