# Supplementary figures and images for: Identification of the global diurnal rhythmic transcripts, transcription factors and time-of-day specific cis elements in Chenopodium quinoa
Source: BMC Plant Biol. 2023 Feb 16;23:96. doi: 10.1186/s12870-023-04107-z (PMC9933291; doi:10.1186/s12870-023-04107-z)

Figure S1

SD

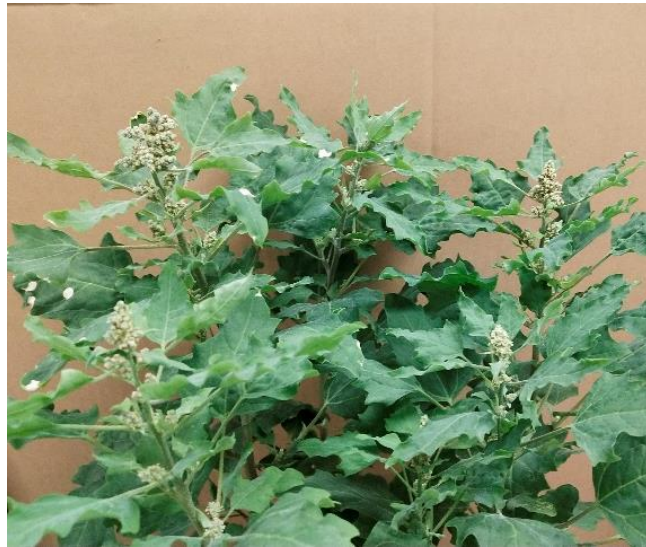

LD

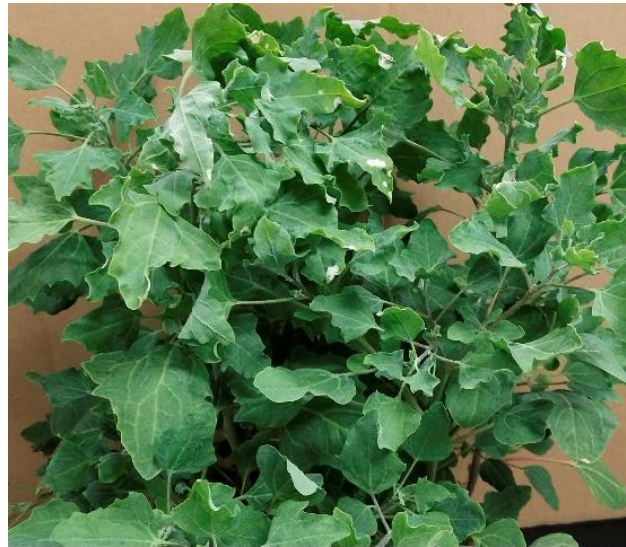

Figure S2

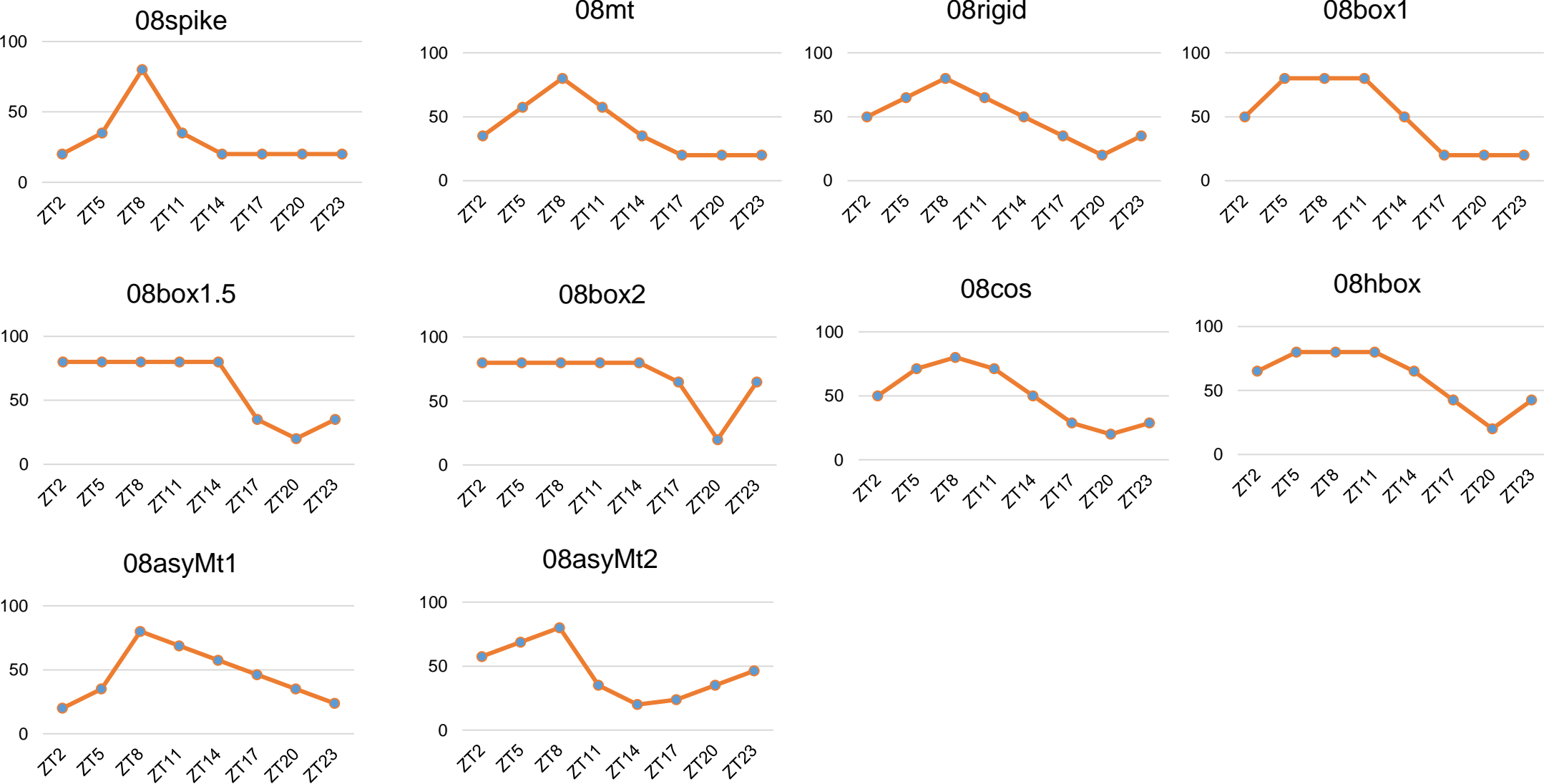

Figure S3

A

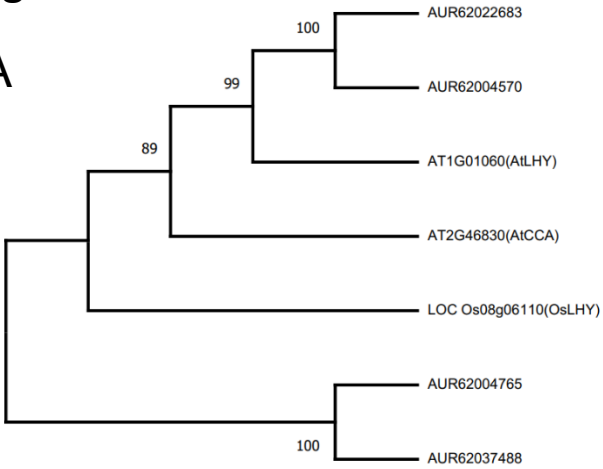

B

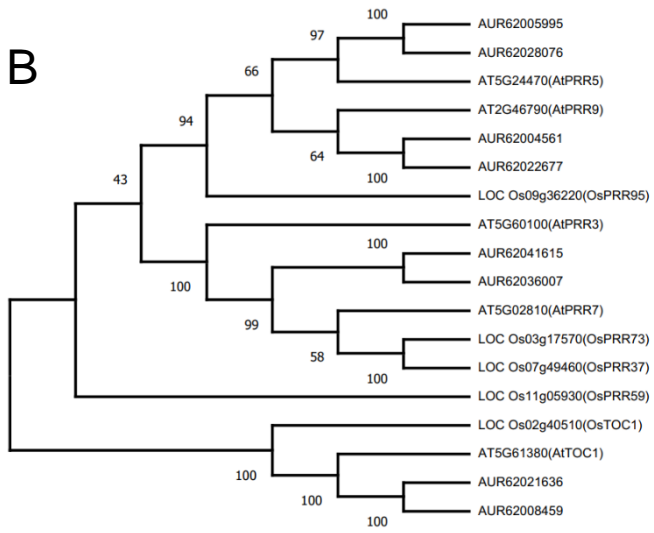

C

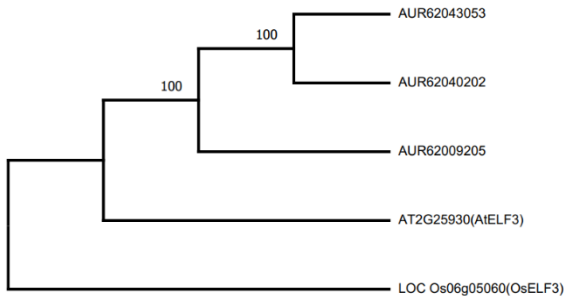

D

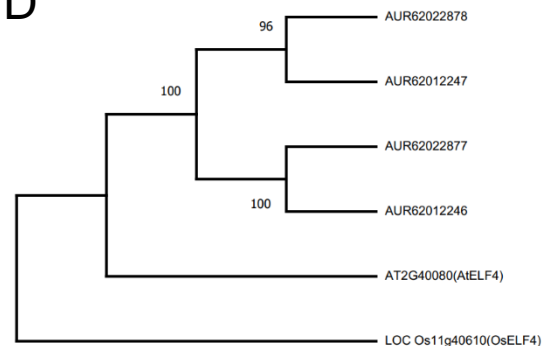

E

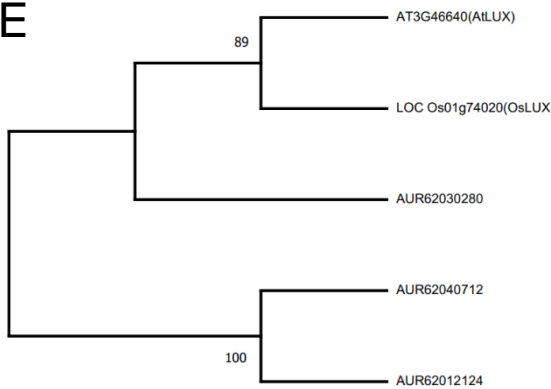

F

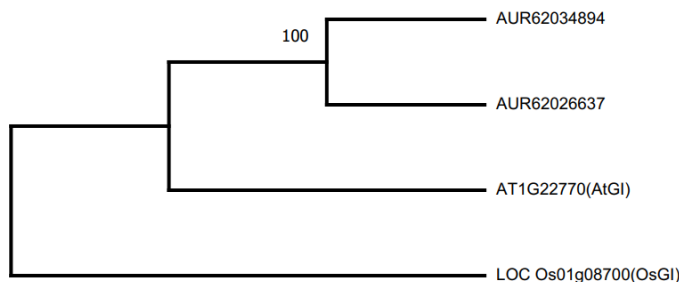

G

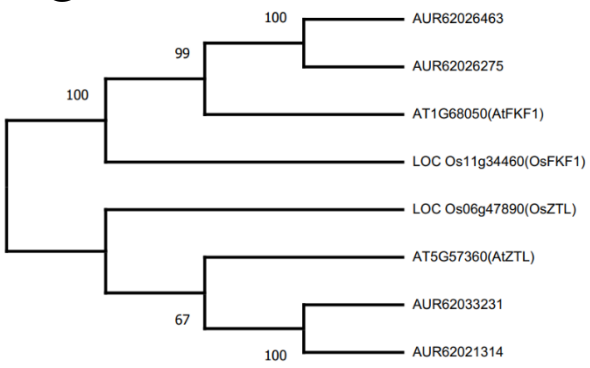

H

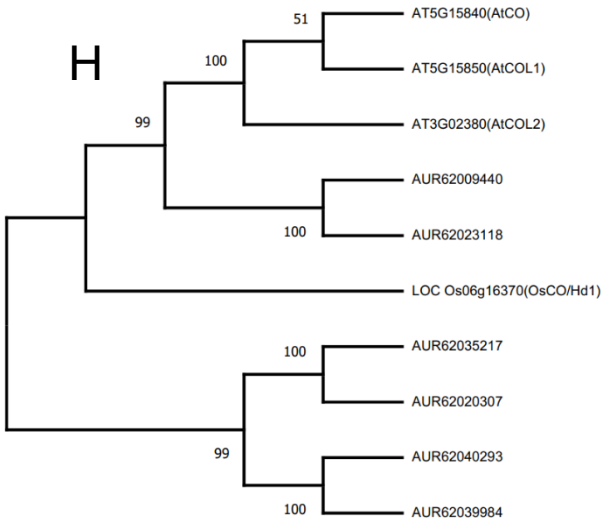

I

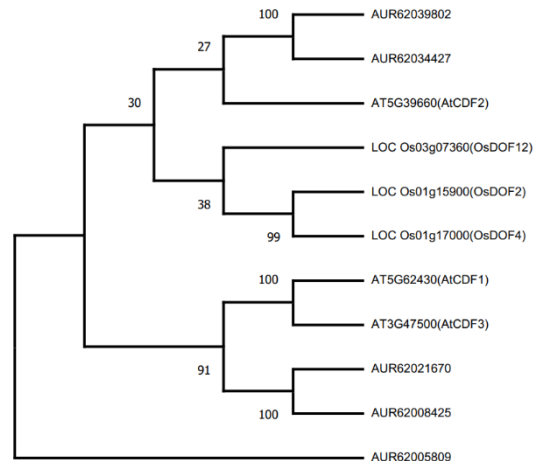

J

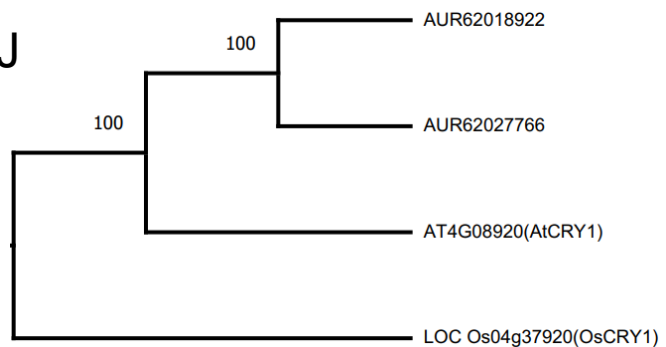

Figure S4

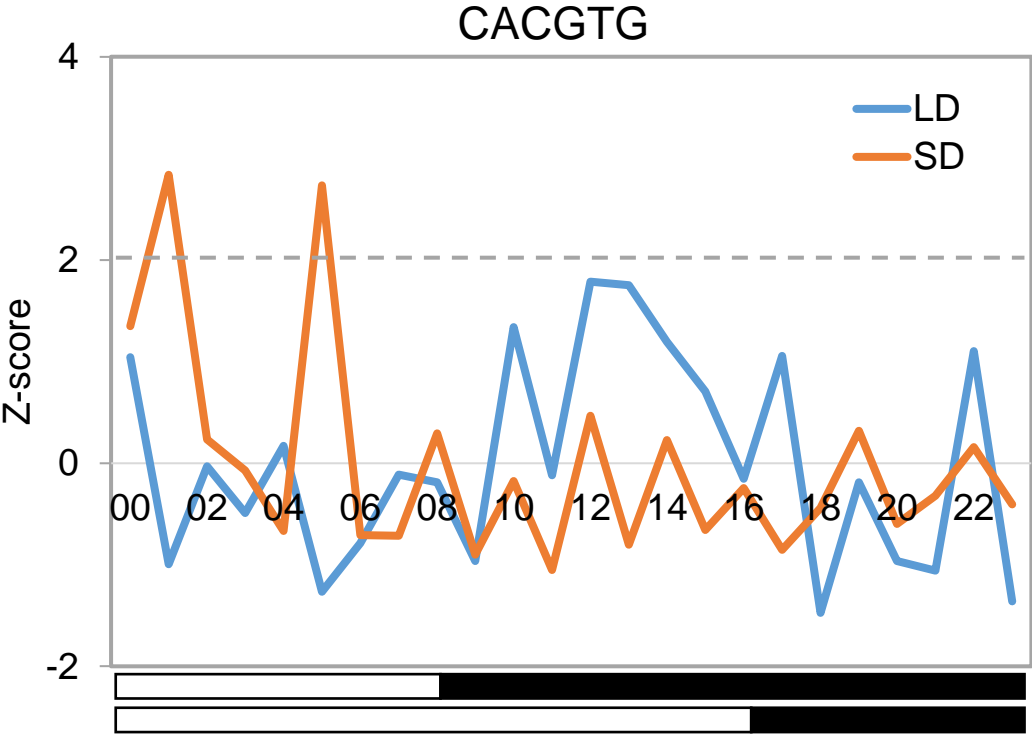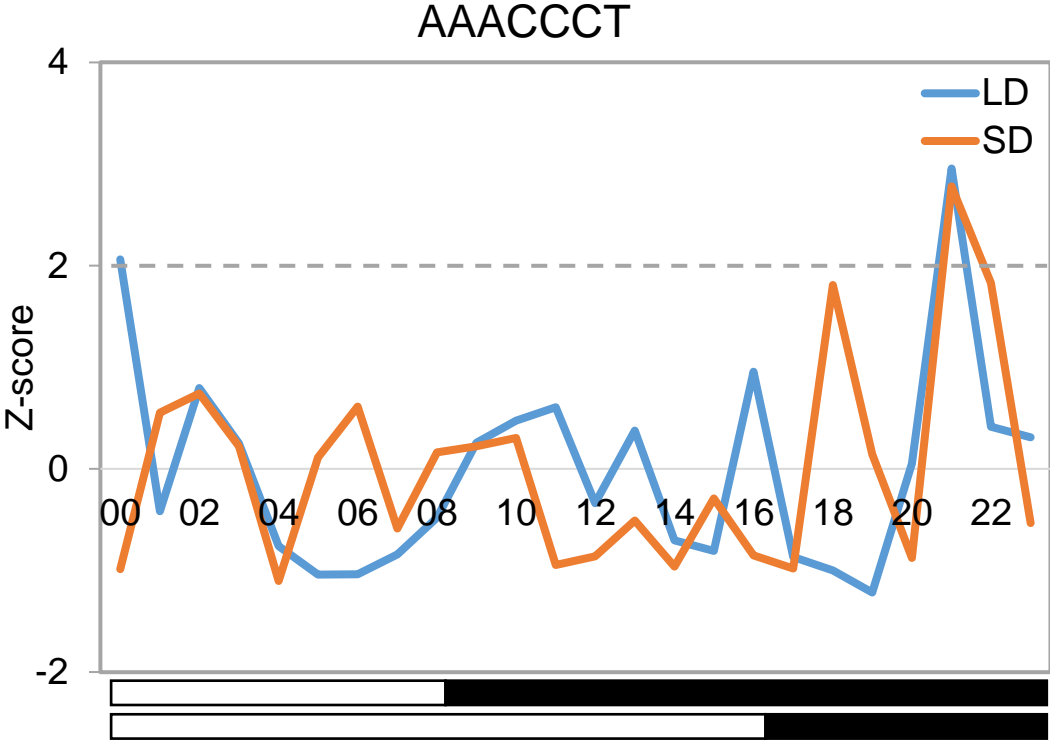

Figure S5

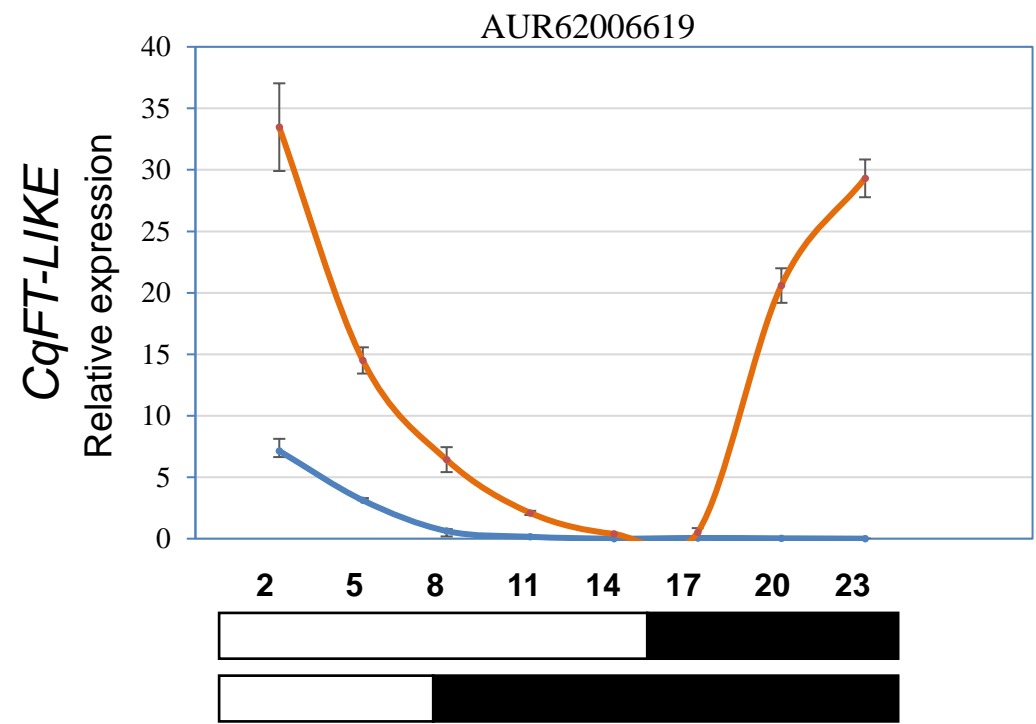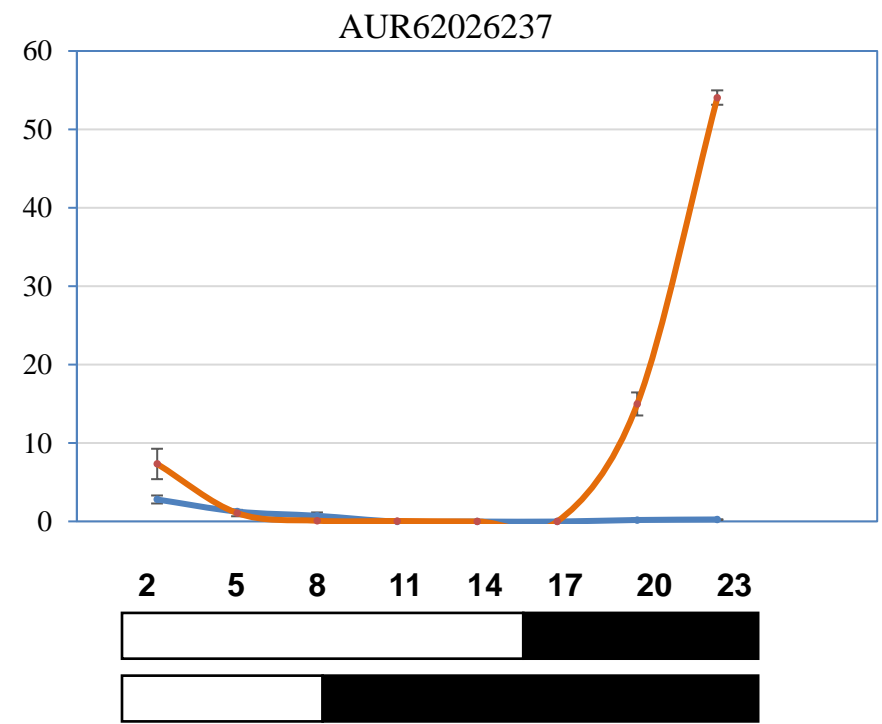

Figure S6

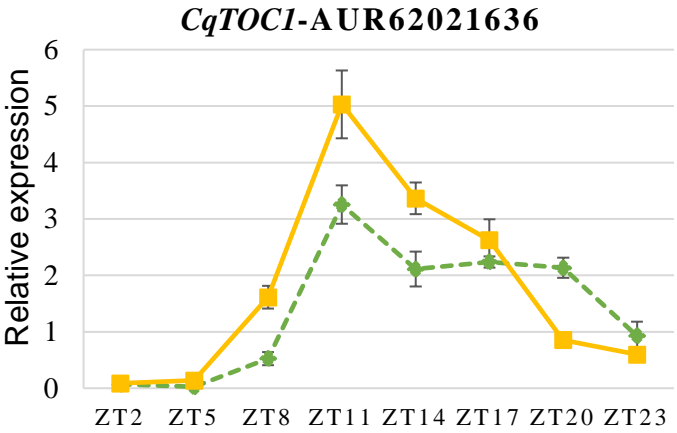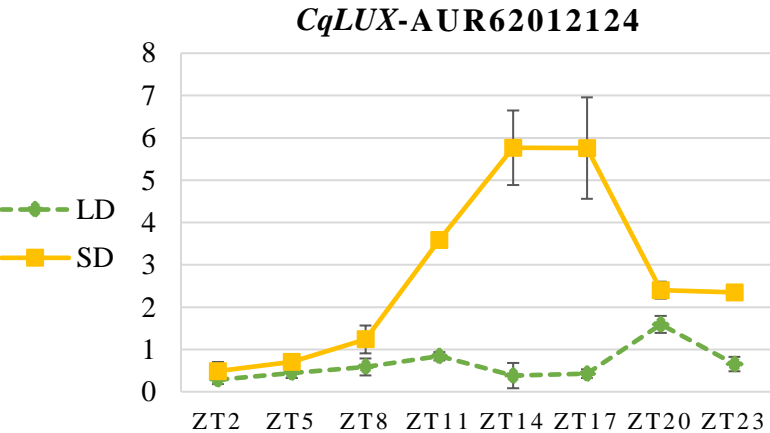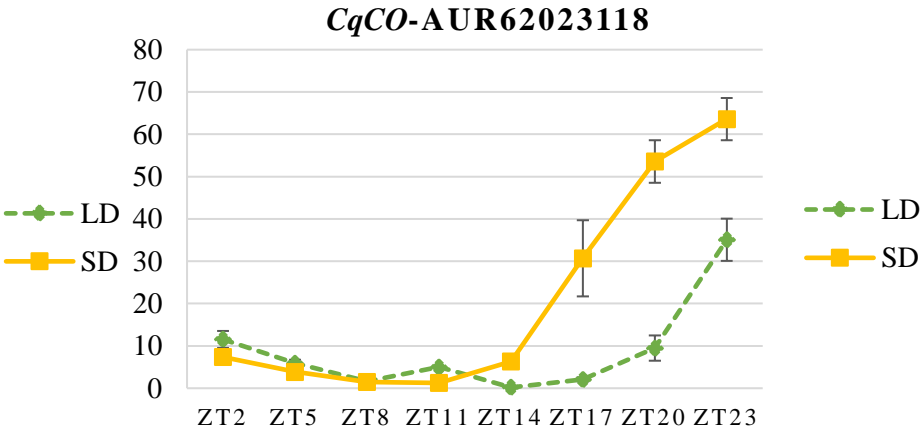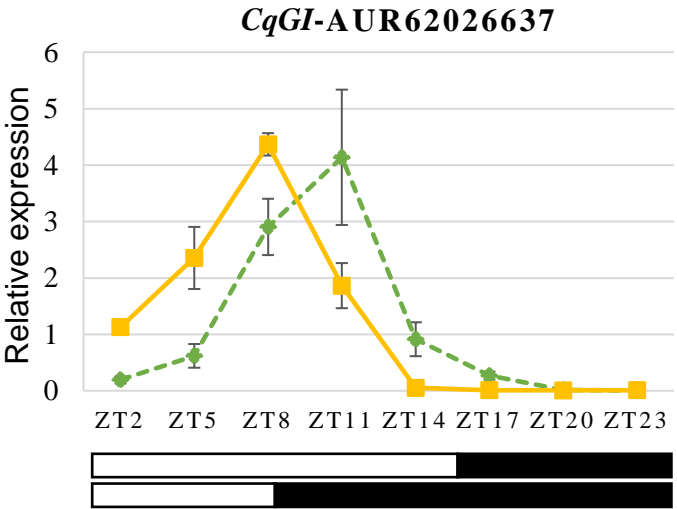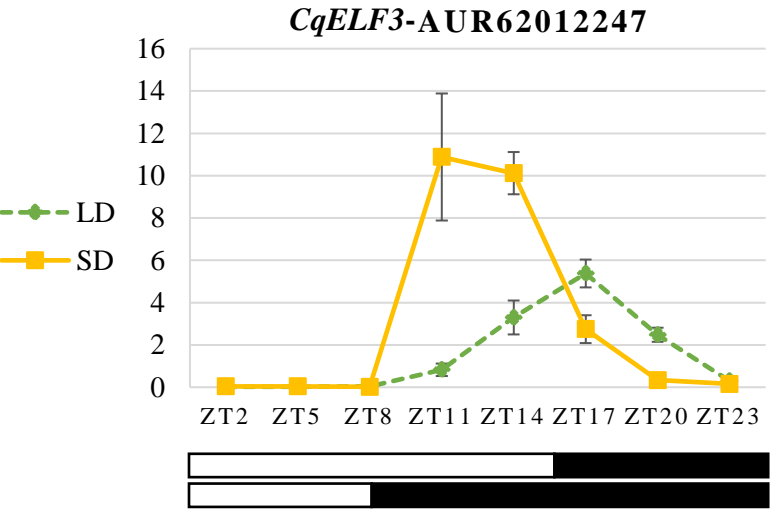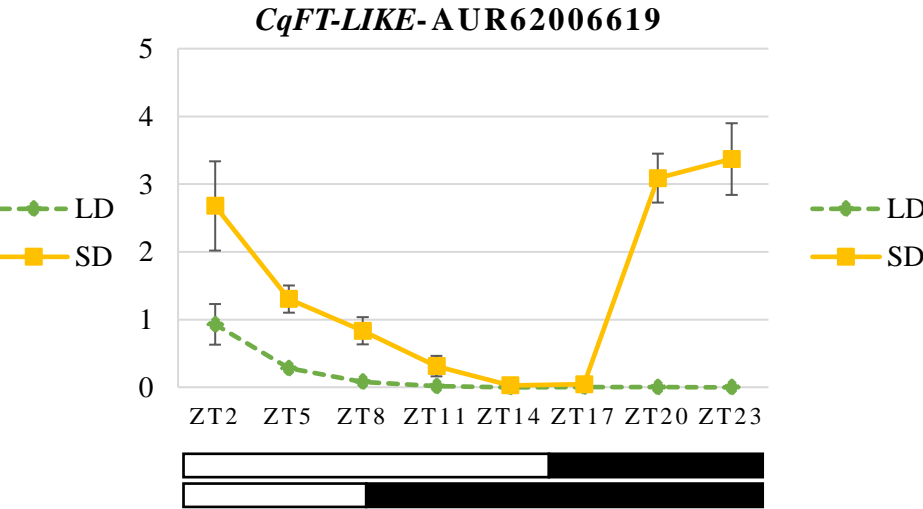

Supplement: Supplementary file 1 — Additional file 1: Figure S1. Phenotypes of quinoa plants grown under SD and LD at 60 days after sowing. The “HL1” plants grown under SD had advanced flowering time than plants grown under LD. Figure S2. Examples of various models shifted to ZT08. The models used to identify diurnal rhythmic transcripts include Asymt1, Asymt2, Box1, Box1.5, Box2, Cos, hBox, Mt, Rigid and Spike. Figure S3. Phylogenic analysis of the circadian clock homologs between Arabidopsis, rice and quinoa. Proteins of (A)LHY-CCA, (B) PRR, (C) ELF3, (D) ELF4, (E) LUX, (F) GI, (G) FKF1-ZTL, (H) CO, (I) CDF and (J) CRY families of Arabidopsis, rice and quinoa were used to construct phylogenic tree using the Neighbor-Joining method. The bootstrap value was 1000 replicates. Figure S4. Frequencies of the representative CCREs under different photoperiods. Z-score profiles of G‐box (CACGTG) and TBX (AAACCCT) in the promoters of rhythmic transcripts of different phase bins under SD and LD were calculated. Figure S5. Expressions of two CqFT-LIKE homologs under SD and LD. mRNA abundances of CqFT-LIKE genes in quinoa leaves under SD were much higher than that under LD. Figure S6. Real-time PCR validation of gene diurnal expression patterns under SD and LD. CqTOC1, CqLUX, CqELF3, CqGI, CqCO and CqFT-LIKE were selected for real-time PCR test. The orange and green curves stand for relative gene expression levels under SD and LD, respectively. The real-time PCR values are mean ± SD (n = 3). [file 12870_2023_4107_MOESM1_ESM.pdf]
